# A comparison of methods for differential expression analysis of RNA-seq data

**DOI:** 10.1186/1471-2105-14-91

**Published:** 2013-03-09

**Authors:** Charlotte Soneson, Mauro Delorenzi

**Affiliations:** 1 Bioinformatics Core Facility, SIB Swiss Institute of Bioinformatics, Lausanne, Switzerland; 2 Département de formation et recherche, Centre Hospitalier Universitaire Vaudois and University of Lausanne, Lausanne, Switzerland

**Keywords:** Differential expression, Gene expression, RNA-seq

## Abstract

**Background:**

Finding genes that are differentially expressed between conditions is an integral part of understanding the molecular basis of phenotypic variation. In the past decades, DNA microarrays have been used extensively to quantify the abundance of mRNA corresponding to different genes, and more recently high-throughput sequencing of cDNA (RNA-seq) has emerged as a powerful competitor. As the cost of sequencing decreases, it is conceivable that the use of RNA-seq for differential expression analysis will increase rapidly. To exploit the possibilities and address the challenges posed by this relatively new type of data, a number of software packages have been developed especially for differential expression analysis of RNA-seq data.

**Results:**

We conducted an extensive comparison of eleven methods for differential expression analysis of RNA-seq data. All methods are freely available within the R framework and take as input a matrix of counts, i.e. the number of reads mapping to each genomic feature of interest in each of a number of samples. We evaluate the methods based on both simulated data and real RNA-seq data.

**Conclusions:**

Very small sample sizes, which are still common in RNA-seq experiments, impose problems for all evaluated methods and any results obtained under such conditions should be interpreted with caution. For larger sample sizes, the methods combining a variance-stabilizing transformation with the ‘limma’ method for differential expression analysis perform well under many different conditions, as does the nonparametric SAMseq method.

## Background

Transcriptome analysis is an important tool for characterization and understanding of the molecular basis of phenotypic variation in biology, including diseases. During the past decades microarrays have been the most important and widely used approach for such analyses, but recently high-throughput sequencing of cDNA (RNA-seq) has emerged as a powerful alternative [1] and it has already found numerous applications [2]. RNA-seq uses next-generation sequencing (NGS) methods to sequence cDNA that has been derived from an RNA sample, and hence produces millions of short reads. These reads are then typically mapped to a reference genome and the number of reads mapping within a genomic feature of interest (such as a gene or an exon) is used as a measure of the abundance of the feature in the analyzed sample [3].

Arguably the most common use of transcriptome profiling is in the search for differentially expressed (DE) genes, that is, genes that show differences in expression level between conditions or in other ways are associated with given predictors or responses. RNA-seq offers several advantages over microarrays for differential expression analysis, such as an increased dynamic range and a lower background level, and the ability to detect and quantify the expression of previously unknown transcripts and isoforms [3-5]. The analysis of RNA-seq data is, however, not without difficulties. Some of these difficulties are inherent to next-generation sequencing procedures. For example, the variation in nucleotide composition between genomic regions implies that the read coverage may not be uniform along the genome. Further, more reads will map to longer genes than to shorter ones with the same expression level. In differential expression analysis, where the genes are tested individually for expression differences between conditions, such ‘within-sample’ biases are usually ignored since they are assumed to affect all samples similarly [3].

Other types of non-uniformities are seen *between* samples in an RNA-seq experiment. First, the *sequencing depths* or *library sizes* (the total number of mapped reads) are typically different for different samples, which means that the observed counts are not directly comparable between samples. Indeed, even in the absence of any true differential expression, if one sample is sequenced to twice the depth of another we expect all the genes to obtain twice as high count in the first sample compared to the second, and we do not want to confuse such effects with true differential expression. The most straightforward way of approaching the different library sizes is to simply rescale or resample the read counts to obtain equal library sizes for all samples. However, such a normalization is generally not enough. The reason is that even if the library sizes are indeed identical, RNA-seq counts inherently represent *relative* abundances of the genes. A few highly expressed genes may contribute a very large part of the sequenced reads in an experiment, leaving only few reads to be distributed among the remaining genes [6]. The presence of the few highly expressed genes thus represses the counts for all other genes, and in comparison to a sample where the reads are more evenly distributed, the latter group of genes may, perhaps incorrectly, seem to have a lower expression which can lead to a lot of genes being falsely called differentially expressed. To account for this difficulty and attempt to make the counts comparable across samples, more complex normalization schemes have been proposed [6-8]. In addition to the library sizes, these procedures also include the estimation of sample-specific *normalization factors* that are used to rescale the observed counts. Using these normalization methods, the sum of the normalized counts across all genes are therefore not necessarily equal between samples (as it would be if only the library sizes were used for normalization), but the goal is instead to make the normalized counts for non-differentially expressed genes similar between the samples. In this study, we use the TMM normalization (trimmed mean of M-values [8]) and the normalization provided in the DESeq package [7]. A comprehensive evaluation of seven different normalization methods was recently performed [9], in which these two methods were shown to perform similarly, and they were also the only ones providing satisfactory results with respect to all metrics used in that evaluation. Still, it is important to keep in mind that even these methods are based on an assumption that most genes are equivalently expressed in the samples, and that the differentially expressed genes are divided more or less equally between up- and downregulation [9].

Microarrays have been used routinely for differential expression analysis for over a decade, and there are well-established methods available for this purpose (such as limma [10]). These methods are not immediately transferable to analysis of RNA-seq data [11], since these are somewhat different from the data obtained from microarrays. The intensities recorded from microarrays are treated as continuous measurements, commonly assumed to follow a log-normal distribution, while the counts from an RNA-seq experiment are non-negative integers and thus inherently follow a discrete distribution. In the methods explicitly developed for differential expression analysis of this type of count data, the Poisson distribution and the Negative Binomial (NB) distribution are the two most commonly used models [7,12-15]. Other distributions, such as the beta-binomial [16], have also been proposed. The Poisson distribution has the advantage of simplicity and has only one parameter, but it constrains the variance of the modeled variable to be equal to the mean. The Negative Binomial distribution has two parameters, encoding the mean and the dispersion, and hence allows modeling of more general mean-variance relationships. For RNA-seq, it has been suggested that the Poisson distribution is well suited for analysis of technical replicates, whereas the higher variability between biological replicates necessitates a distribution incorporating overdispersion, such as the Negative Binomial [6,17]. Instead of using integer counts directly, some software packages represent RNA-seq data by transformed quantities such as RPKM (Reads Per Kilobase per Million mapped reads) [1] or the related FPKM (Fragments Per Kilobase per Million mapped reads) [18]. The goal of such transformations is to normalize the counts with respect to the differing library sizes and with respect to the length of the transcripts, since a long transcript is expected to obtain more reads than a short transcript with the same expression level. Other normalization strategies can be employed to handle other biases, arising for example from the variable GC content of the reads. After transformations such as these, the resulting values are no longer integer counts, which means that they should not be plugged into count-based methods for differential expression analysis. Among the methods evaluated in this study, only the non-parametric ones would thus be suitable also for RPKM values. Other software, such as Cufflinks/Cuffdiff [18], provide an integrated analysis pipeline from the aligned reads to the differential expression results, where the inference is based on FPKM values.

The field of differential expression analysis of RNA-seq data is still in its infancy and new methods are continuously being presented. So far, there is no general consensus regarding which method performs best in a given situation and few extensive comparisons between the proposed methods have been published. In a recent paper [19], four parametric methods were compared in terms of their ability to discriminate between truly differentially expressed (DE) and truly non-DE genes, under different simulation conditions. The authors also compared the overlap between the sets of DE genes found by the different methods in a real data set. Another recent study [20] evaluated the impact of increasing sequencing depth on the ability to detect DE genes and contrasted this with the benefits of increasing the sample size, and the latter were found to be considerably larger. In [21], the authors presented a case study on *Saccharomyces cerevisiae*, comparing the results obtained by several differential expression analysis methods for RNA-seq with each other and with results obtained from microarrays, and reported a generally good agreement between the different methods.

In the present paper we conduct a comparison of eleven methods, developed for differential expression analysis of RNA-seq data, under different experimental conditions. Among the eleven methods, nine model the count data directly, while the remaining two transform the counts before applying a traditional method for differential expression analysis of microarray data. The study is confined to methods that are implemented and available within the R framework [22] and that are applicable to count matrices (containing the count for each of a number of genes or other genomic features of interest in each of a number of samples). Several methods for obtaining such a matrix from the raw sequence data exist, but a comprehensive evaluation of these are outside the scope of the present study. We further focus on finding genes that are differentially expressed between two conditions only, since this is arguably the most commonly encountered application. Moreover, it is supported by all evaluated methods, although most methods allow also more complex experimental designs (see further in the Materials and Methods section).

## Results and discussion

Eleven methods for differential expression analysis of RNA-seq data were evaluated in this study. Nine of them work on the count data directly: DESeq [7], edgeR [23], NBPSeq [15], TSPM [13], baySeq [14], EBSeq [24], NOISeq [25], SAMseq [26] and ShrinkSeq [27]. The remaining two combine a data transformation with limma [10] for differential expression analysis, and we will refer to them as voom(+limma) [10] and vst(+limma) [7,10]. More detailed descriptions of the methods can be found in the Materials and Methods section and in the respective original publications.

The methods were evaluated mainly based on synthetic data, where we could control the settings and the true differential expression status of each gene. Details regarding the different simulation studies can be found in the Materials and Methods section. As the baseline (simulation studies abbreviated ‘*B*’), we simulated all counts using Negative Binomial distributions, with mean and dispersion parameters estimated from real data. In these simulations, the dispersions in both conditions were assumed to be identical. Note that this does not imply that the variances are the same in the two conditions, since the variance depends also on the mean. We also evaluated the robustness of the methods against variations in the distribution of the input data, by instead imposing a Poisson distribution for the counts for some of the genes (simulation studies denoted ‘*P*’), or including outliers with abnormally high counts (simulation studies denoted ‘*S*’ and ‘*R*’). The outliers were introduced in two different ways. For the ‘single’ outlier simulation studies (denoted ‘*S*’), we selected 10% of the genes, and for each of these genes we selected a single sample for which we multiplied the observed count with a randomly generated factor between 5 and 10. For the ‘random’ outlier simulation studies (denoted ‘*R*’), we considered each observed count independently, and with probability 0.05 we multiplied it with a randomly generated factor between 5 and 10.

The total number of genes in each simulated data set was 12,500, and the number of differentially expressed (DE) genes was set to either 0, 1,250 or 4,000. We also varied the composition of the DE genes, that is, the fraction of DE genes that were up- and downregulated, respectively, in one condition compared to the other. Finally, we evaluated the effect of varying the sample size, from 2 to 5 or 10 samples per condition. These sample sizes were chosen to reflect a wide range of experimental settings. Since, however, most current RNA-seq experiments exhibit small sample sizes and the choice in the experimental design is often between two or three samples per condition, we also performed some comparisons with 3 samples per condition. These comparisons, contrasted with the results from 2 and 5 samples per condition, are given in the supplementary material (Additional file 1). In the supplementary material we also present some results obtained for data sets where the dispersion parameters were different between the two conditions.

In addition to the simulated data, we compared the methods based on their performance for three real RNA-seq data set. The results from one of these data sets are shown in the main article, and the remaining two are discussed in the supplementary material (Additional file 1).

Using the synthetic data, we studied the following aspects of the methods under different experimental conditions:

•The ability to rank truly DE genes ahead of non-DE genes. This was evaluated in terms of the area under a Receiver Operating Characteristic (ROC) curve (AUC), as well as in terms of false discovery curves, depicting the number of false detections encountered while going through the list of genes ranked according to the evidence for differential expression.

•The ability to control type I error rate and false discovery rate at an imposed level. This was evaluated by computing the observed type I error and the true false discovery rate, respectively, among the genes called differentially expressed at given significance levels.

•The computational time requirement for running the differential expression analysis. These results are given in the supplementary material (Additional file 1).

For the real RNA-seq data we compared the collections of genes called DE by the different methods, both in terms of their individual cardinalities and in terms of their overlaps. We also studied the concordance of the gene rankings obtained by the different methods.

### Discrimination between DE and non-DE genes

We first evaluated to what extent the eleven considered methods were able to discriminate between truly DE genes and truly non-DE ones. We computed a score for each gene and each method, which allowed us to rank the genes in order of significance or evidence for differential expression between the two conditions. For the six methods providing nominal p-values (edgeR, DESeq, NBPSeq, TSPM, voom+limma, vst+limma), we defined the score as 1 - *p*_*nom*_. For SAMseq we used the absolute value of the averaged Wilcoxon statistic as the ranking score, and for baySeq, EBSeq and ShrinkSeq we used the estimated posterior probability of differential expression or, equivalently in terms of ranking, 1 - BFDR, where BFDR denotes the estimated Bayesian False Discovery Rate [28] (see Materials and Methods for more information about the different methods). For NOISeq, we used the statistic *q*_*NOISeq*_ (see Materials and Methods). All these scores are two-sided, that is, they are not affected by the direction of differential expression between the two conditions. Given a threshold value for such a score, we may thus choose to call all genes with scores exceeding the threshold DE, and correspondingly all genes with scores below the threshold are called non-DE. Considering the genes that were simulated to be DE as the true positive group and the remaining genes as the true negative group, we computed the false positive rate and the true positive rate for all possible score thresholds and constructed a ROC (Receiver Operating Characteristic) curve for each method. The area under the ROC curve (AUC) was used as a measure of the overall discriminative performance of a method, that is, the overall ability to rank truly DE genes ahead of truly non-DE ones.

**Figure 1:**
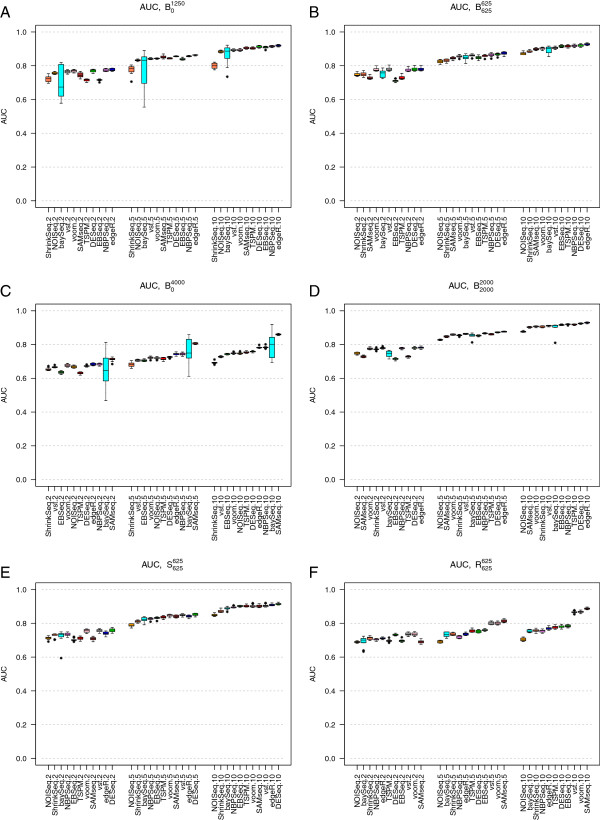
**Area under the ROC curve (AUC).** Area under the ROC curve (AUC) for the eleven evaluated methods, in simulation studies $B_{0}^{1250}$ (panel **A**), $B_{625}^{625}$ (panel **B**), $B_{0}^{4000}$ (panel **C**), $B_{2000}^{2000}$ (panel **D**), $S_{625}^{625}$ (panel **E**) and $R_{625}^{625}$ (panel **F**). The boxplots summarize the AUCs obtained across 10 independently simulated instances of each simulation study. Each panel shows the AUCs across three sample sizes (|S_1_| = |S_2_| = 2, 5 and 10, respectively, signified by the last number in the tick labels). The methods are ordered according to their median AUC for the largest sample size. When all DE genes were regulated in the same direction, increasing the number of DE genes from 1,250 (panel **A**) to 4,000 (panel **C**) impaired the performance of all methods. In contrast, when the DE genes were regulated in different directions (panels **B** and **D**), the number of DE genes had much less impact. The variability of the performance of baySeq was much higher when all genes were regulated in the same direction (panels **A** and **C**) compared to when the DE genes were regulated in different directions (panels **B** and **D**). Including outliers (panels **E** and **F**) decreased the AUC for most methods (compare to panel **B**), but less so for the transformation-based methods (voom+limma and vst+limma) and SAMseq.

Under baseline conditions, and when only 10% of the genes were simulated to be DE (simulation studies $B_{0}^{1250}$ and $B_{625}^{625}$), the composition of the set of DE genes (in terms of up- or downregulation) had only a minor impact on the gene ranking accuracy for most methods (compare Figures 1A and 1B). When almost one third of the genes were DE (simulation studies $B_{0}^{4000}$ and $B_{2000}^{2000}$), the effect of the composition of the set of DE genes became more dramatic. Now, the performances of all methods were considerably worse when all DE genes were upregulated in S_2_ compared to S_1_ than when some genes were upregulated and some were downregulated (compare Figures 1C and 1D). A possible explanation for this effect is that the normalization factors, which are designed to account for this type of varying count distributions, are not able to estimate the effect to a full extent which leads to a lot of false positive results, mixed with the true positives. Notably, SAMseq, which uses a resampling strategy to equalize library sizes and thus implicitly assumes that all normalization factors are equal, showed the best performance in simulation study $B_{0}^{4000}$, where all the 4,000 DE genes were upregulated in condition S_2_ compared to condition S_1_ (Figure 1C).

For the largest sample sizes (5 or 10 samples per condition) and when there were both up- and downregulated genes, all methods performed similarly in terms of the AUC. All methods performed better for large sample sizes. TSPM and EBSeq showed the strongest sample size dependencies among the methods, followed by SAMseq and baySeq. For the smallest sample size (2 samples per condition), the best results were generally obtained by DESeq, edgeR, NBPSeq, voom+limma and vst+limma.

When all DE genes were upregulated in condition S_2_ compared to condition S_1_ (Figures 1A and 1C), we saw a high variability in the results obtained by baySeq. This variability was reduced when the DE genes were regulated in different directions (Figures 1B and 1D).

We chose to evaluate the effect of introducing non-overdispersed genes or outliers under the settings of simulation study $B_{625}^{625}$ (Figure 1B). When the fraction of genes following a Poisson distribution was increased from 0 to 50% (simulation study $P_{625}^{625}$) the AUC increased, especially for the smallest sample size (Additional file 1: Figure S17, compare to Figure 1B). Outliers with abnormally high counts reduced the AUC slightly for all methods, but less for the transformation-based methods (vst+limma and voom+limma) and SAMseq than for the other methods (Figures 1E and 1F).

**Figure 2:**
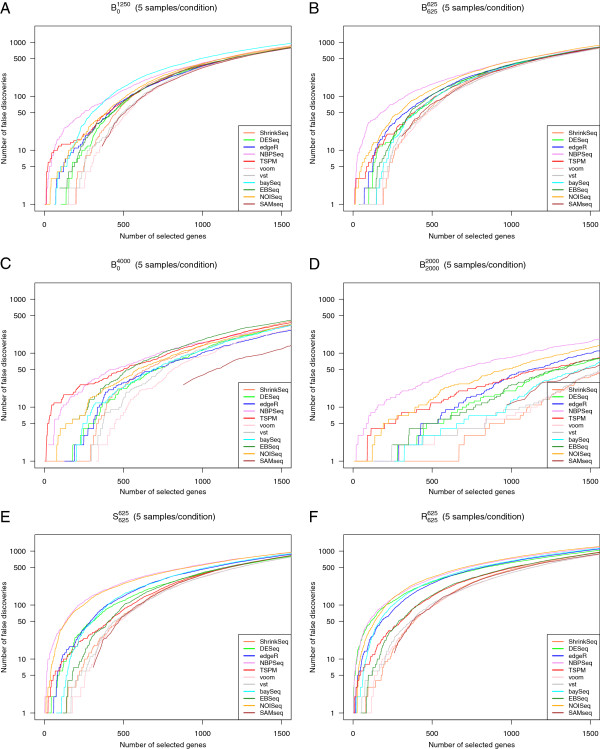
**False discovery curves.** Representative false discovery curves, depicting the number of false positives encountered among the T top-ranked genes by the eleven evaluated methods, for T between 0 and 1,500. In all cases, there were 5 samples per condition. **A**: Simulation study $B_{0}^{1250}$. **B**: Simulation study $B_{625}^{625}$. **C**: Simulation study $B_{0}^{4000}$**D**: Simulation study $B_{2000}^{2000}$. **E**: Simulation study $S_{625}^{625}$**F**: Simulation study $R_{625}^{625}$. Some of the curves do not pass through the origin, since many genes obtained the same ranking score and had to be called simultaneously.

While the AUC provides an overall measure of the ability to rank truly DE genes ahead of truly non-DE genes, it does not immediately tell us if the deviation from a perfect discrimination is mainly due to false positives or false negatives. We therefore also constructed false discovery curves, depicting the number of false discoveries encountered as the total number of discoveries increased (that is, as the significance threshold for the ranking score was changed). Figure 2 shows representative false discovery curves for the same simulation studies that were considered in Figure 1, with 5 samples per condition. In the supplementary material (Additional file 1) we show corresponding curves for 2 and 10 samples per condition, respectively (Additional file 1: Figures S18-S19). Given that we are most interested in the genes showing the strongest evidence of differential expression, we confined the analysis to the 1,500 top-ranked genes for each method. We noted that although NBPSeq was among the best methods in terms of the overall ranking (the highest AUC, see Figure 1), it had problems with false discoveries among the very top-ranked genes under many simulation settings. Indeed, while the total number of false discoveries among the 1,500 top-ranked genes were in parity with many other methods, there were often some false discoveries ranked very near the top by NBPSeq. TSPM and NOISeq also tended to rank some truly non-DE genes in the very top. For simulation study $P_{625}^{625}$, where half of the genes were generated according to a Poisson distribution, the performance of TSPM was improved and fewer non-DE genes were ranked near the top (Additional file 1: Figure S17). Overall, the best performance, in terms of ranking mainly true positives in the very top, was obtained with the transformation-based methods (voom+limma and vst+limma) and ShrinkSeq. SAMseq also performed well, but returned the same (top) score for many genes, both truly DE and truly non-DE.

Larger sample sizes led to considerably fewer false positives found among the top-ranked genes (compare Figure 2 to Additional file 1: Figures S18 and S19). Actually, as seen by comparing Additional file 1: Figure S18 to Additional file 1: Figures S10(b) and 11(b), already increasing the number of samples per condition from 2 to 3 provided a tangible improvement.

### Control of type I error rate

**Figure 3:**
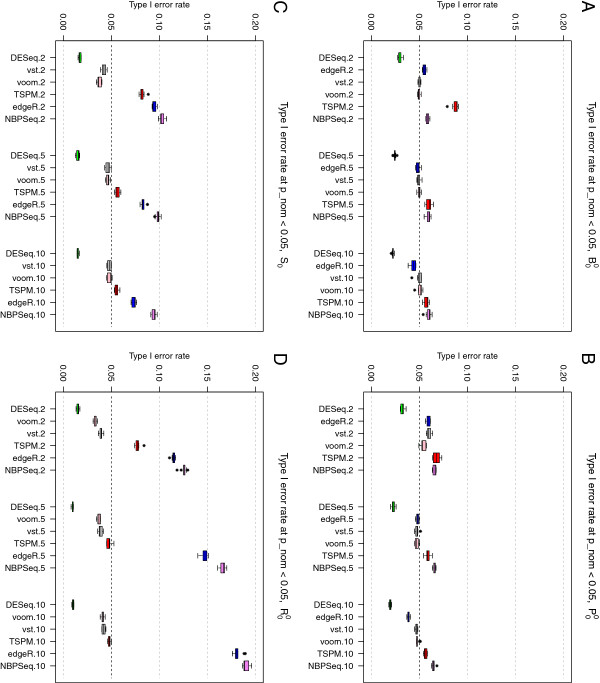
**Type I error rates.** Type I error rates, for the six methods providing nominal p-values, in simulation studies $B_{0}^{0}$ (panel **A**), $P_{0}^{0}$ (panel **B**), $S_{0}^{0}$ (panel **C**) and $R_{0}^{0}$ (panel **D**). Letting some counts follow a Poisson distribution (panel **B**) reduced the type I error rates for TSPM slightly but had overall a small effect. Including outliers with abnormally high counts (panels **C** and **D**) had a detrimental effect on the ability to control the type I error for edgeR and NBPSeq, while DESeq became slightly more conservative.

Next, we evaluated the six methods returning nominal p-values (edgeR, DESeq, NBPSeq, TSPM, voom+limma and vst+limma) in terms of their ability to control the type I error at a pre-specified level in the absence of any truly DE genes. Under baseline conditions (simulation study $B_{0}^{0}$) and using a nominal p-value cutoff of 0.05, all six methods performed quite well and in many cases called around 5% of the genes differentially expressed (Figure 3A). NBPSeq and TSPM found the highest number of false positives and DESeq was the most conservative among the six methods. This is concordant with the findings in a previous study [19] where the type I error rate control of edgeR, DESeq and NBPSeq were compared. The strongest dependence on sample size was seen for TSPM, which performed poorly for the smallest sample size (two samples per condition), but in parity with the other methods for the larger sample sizes. A slight reduction in type I error rate with increasing sample size was seen also for edgeR and DESeq while the performance of the transformation-based approaches and NBPSeq were less sample-size dependent.

The results stayed largely similar when we let the counts for half of the genes be Poisson distributed (simulation study $P_{0}^{0}$, Figure 3B), but for the smallest sample size we noted a reduction of the type I error rate for TSPM and an increase of the type I error rate for the transformation-based methods and NBPSeq. Introducing ‘single’ outliers (simulation study $S_{0}^{0}$) had a considerable effect on the type I error of the three methods that are explicitly modeling the counts using a Negative Binomial distribution (edgeR, DESeq and NBPSeq). Under these conditions, the type I error rates of NBPSeq and edgeR increased substantially, while DESeq instead became even more conservative (Figure 3C). The type I error rates of the transformation-based methods and the TSPM were less affected, but tended to decrease rather than increase following the introduction of outliers. Similar effects, but even more pronounced, were noted when we instead introduced ‘random’ outliers (simulation study $R_{0}^{0}$) Figure 3D, see the Materials and Methods section for a more extensive explanation of the different types of outliers). If these outliers were instead introduced by dividing the counts by a random factor between 5 and 10 (instead of multiplying with this factor), the results were largely similar to those from the baseline study (without outliers), except for a slight reduction of the type I error rate for NBPSeq and edgeR (data not shown). In Additional file 1 (Additional file 1: Figures S20 and S21), we show representative p-value distributions under the different simulation settings. In these figures, we note that even when all null hypotheses are true, the p-values are not always uniformly distributed. Specifically, some methods (edgeR, DESeq and NBPSeq) exhibit an excess of large p-values. This has been observed also in previous studies and has been attributed to the use of exact tests based on discrete probability distributions [20]. Since the total number of reads mapping to the different genes is very different, the null distribution of p-values will be a mixture of a large number of different discrete distributions [29].

### Control of the false discovery rate

Next, we examined whether setting a significance threshold for the adjusted p-value (or an FDR threshold) indeed controlled the false discovery rate at the desired level. We put the FDR threshold at 0.05, and calculated the true false discovery rate as the fraction of the genes called significant at this level that were indeed false discoveries. Since NOISeq does not return a statistic that is recommended to use as an adjusted p-value or FDR estimate, it was excluded from this evaluation. For baySeq, EBSeq and ShrinkSeq, we imposed the desired threshold on the Bayesian FDR [28].

**Figure 4:**
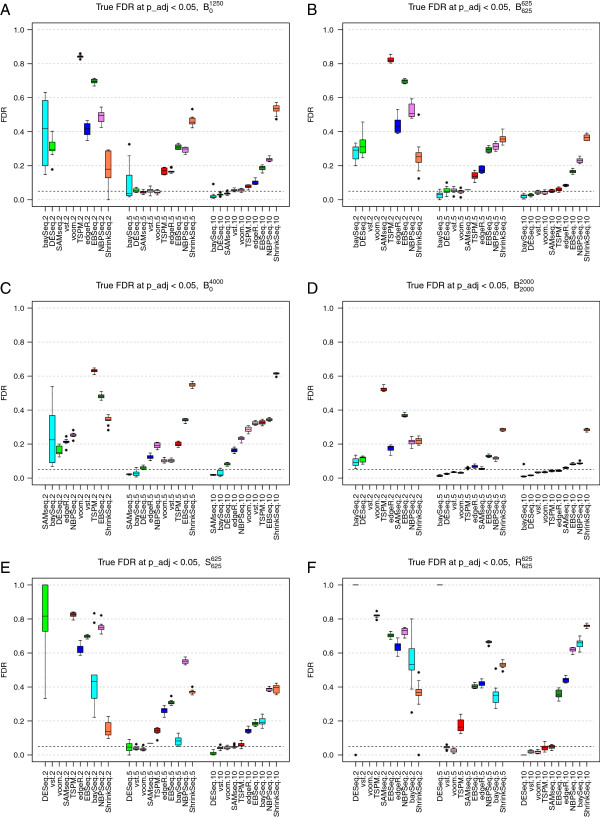
**True false discovery rates.** True false discovery rates (FDR) observed for an imposed FDR threshold of 0.05, for the nine methods returning adjusted p-values or FDR estimates, in simulation studies $B_{0}^{1250}$ (panel **A**), $B_{625}^{625}$ (panel **B**), $B_{0}^{4000}$ (panel **C**) $B_{2000}^{2000}$, (panel **D**), $S_{625}^{625}$ (panel **E**) and $R_{625}^{625}$ (panel **F**). With only two samples per condition, three of the methods (vst+limma, voom+limma and SAMseq) did not call any DE genes, and the FDR was considered to be undefined.

As above, when only 10% of the genes were DE, the direction of their regulation had little effect on the false discovery rate (simulation studies $B_{0}^{1250}$ and $B_{625}^{625}$, compare Figures 4A and 4B). The main difference between the two settings was seen for ShrinkSeq, whose FDR control was worse when all genes were regulated in the same direction. The high false discovery rate seen for ShrinkSeq can possibly be reduced by setting a non-zero value for the fold change threshold defining the null model. Also the variability of the baySeq performance was considerably reduced when there were both up- and downregulated genes among the DE ones. For the largest sample size (10 samples per group), ShrinkSeq, NBPSeq, EBSeq, edgeR and TSPM often found too many false positives. The remaining methods were essentially able to control the false discovery rate at the desired level under these conditions. A possible explanation for the high false discovery rates of NBPSeq is that the dispersion parameters, and thereby also the variances, are understimated for many genes which implies that the significance of these genes are overestimated. When the sample size was decreased, all methods except ShrinkSeq performed considerably worse in terms of FDR control, and with only two samples per group, all methods were far from controlling the true false discovery rate at the desired level. TSPM was most heavily affected by the decreasing sample size, in terms of increasing FDR, which is in agreement with previous observations [19]. With only 2 samples per condition, neither SAMseq nor the two transformation-based methods called any genes significantly DE. As for the false discovery curves above, already an increase in sample size from 2 to 3 samples per condition improved the FDR for many of the methods, in particular DESeq and baySeq, and both transformation-based methods were able to find differentially expressed genes (with reasonably low FDR) with 3 samples per condition (Additional file 1: Figures S10(c) and S11(c)).

When the DE genes were regulated in different directions, increasing the number of DE genes from 1,250 to 4,000 improved the ability to control the FDR (simulation study $B_{2000}^{2000}$ Figure 4D, compare to Figure 4B). Conversely, when all DE genes were regulated in the same direction, increasing the number of DE genes impaired the ability to control the FDR especially for the largest sample sizes (simulation study $B_{0}^{4000}$, Figure 4C, compare to Figure 4A). When outliers with extremely high counts were introduced (simulation studies $S_{625}^{625}$ and $R_{625}^{625}$ the FDRs of baySeq, NBPSeq and edgeR, which are all based on a Negative Binomial distribution, were considerably increased. The transformation-based methods were less affected and controlled the FDR under these conditions as well (Figures 4E and 4F). Also the FDRs of SAMseq and TSPM were largely unaffected by the inclusion of outliers.

In a practical situation, we are not only interested in keeping the rate of false discoveries low, but also to actually be able to find the true positives. Therefore, we also computed the true positive rate (the fraction of truly DE genes that were found to be significant) among the genes that were called significant at a FDR threshold of 0.05. In general, DESeq and baySeq tended to give the lowest number of true positives (Additional file 1: Figure S22). This should be viewed in relation to Figure 4, where it was shown that these methods often also gave low fractions of false discoveries. The other two methods that are based on the NB model, edgeR and NBPSeq, as well as ShrinkSeq, in which we used a zero-inflated NB model, returned more true positives but at the price of a higher false discovery rate. The non-parametric SAMseq method gave high true positive rates across all simulation settings, seemingly without an accompanying high false discovery rate. However, for the smallest sample sizes this method did not find any significantly differentially expressed genes at all which is not surprising due to its non-parametric nature and reliance on sample permutations. The true positive rate of EBSeq was largely unaffected by the sample size, but the false discovery rate decreased as sample size increased.

As expected, increasing the expression difference between the two conditions (*w*_*g*_, see Materials and Methods) improved the ability to detect truly DE genes and reduced the observed false discovery rate, in a concordant manner for all methods (data not shown). When the dispersions in the two conditions were different, we observed an increased FDR for the majority of the methods (Additional file 1: Figure S12(c), compare to Figure 4B).

### Real RNA-seq data from two mouse strains

In addition to the synthetic data set, we also analyzed an RNA-seq data set from 21 mice, 10 of the C57BL/6J strain and 11 of the DBA/2J strain [30]. After filtering out genes for which the total count across the 21 mice was less than 10, the data set contained 11,870 genes. We applied the eleven methods to find genes that showed differential expression between the two mouse strains. All genes found to be DE at a FDR or Bayesian FDR threshold of 0.05 were considered significantly DE. It is not clear how to set a threshold for the q-value returned by NOISeq to be comparable with the FDR estimate or adjusted p-value from the other methods, and hence NOISeq was excluded from most of the subsequent analysis.

**Figure 5:**
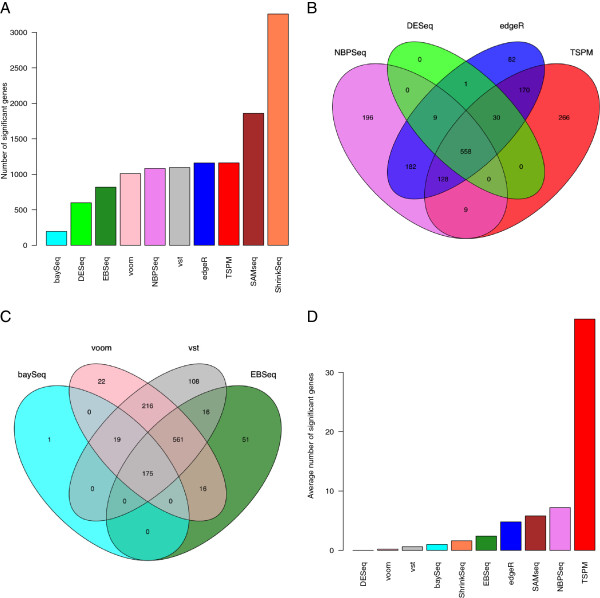
**Analysis of the Bottomly data set. A**: The number of genes found to be significantly DE between the two mouse strains in the Bottomly data set. **B-C**: Overlap among the set of DE genes found by different methods. **D**: The average number of genes found to be significantly DE genes when contrasting two subsets of mice from the same strain, in which case we expect no truly DE genes.

First, we compared the number of DE genes found by each method (Figure 5A). The highest number of DE genes was found by ShrinkSeq, while baySeq returned relatively few. As can be seen in Figure 5A, TSPM, edgeR, NBPSeq and the two transformation-based methods found approximately the same number of DE genes. Next, we studied the overlap between the sets of genes called DE by different methods. Figure 5B shows the overlap between the sets of DE genes found by edgeR, DESeq, NBPSeq and TSPM (only four methods were included in order to make the Venn diagram interpretable). From this figure, we noted that the DE genes found by DESeq were to a large extent found also by edgeR, NBPSeq and TSPM (recall that the three latter found more DE genes). In contrast, both edgeR, NBPSeq and TSPM found a fair amount of ‘unique’ DE genes, that were not shared with the other methods. Figure 5C shows the corresponding comparison for baySeq, EBSeq and the two transformation-based methods. The DE genes found by voom+limma essentially formed a subset of the slightly larger set of DE genes found by vst+limma. Similarly, many of the DE genes found by baySeq were also found by EBSeq, and the DE genes found by EBSeq were to a large extent found also by the transformation-based methods. The set of DE genes found by SAMseq and ShrinkSeq, finally, contained a large part of the genes found by all the other methods. Table 1 shows the overlap between the collections of differentially expressed genes for each pair of methods. To characterize the sets of genes preferentially called DE by the different methods, we marked the DE genes in an MA-like plot (Additional file 1: Figure S23). These results showed clearly that for all methods, a higher fold change was needed for significance for the genes with low average expression. baySeq seemed to require a higher fold change than the other methods across all expression levels, and did not call any highly expressed genes DE. In contrast, SAMseq and ShrinkSeq required a lower fold change for calling highly expressed genes DE, while the threshold for lowly expressed genes was similar to that from the other methods. The low fold change required for highly expressed genes may potentially compromise the biological significance of some of the findings from SAMseq and ShrinkSeq and may necessitate the inclusion of an additional fold change threshold.

**Table 1:** The number of shared differentially expressed genes found by the different methods for the Bottomly data set

	**ShrinkSeq**	**DESeq**	**edgeR**	**NBPSeq**	**TSPM**	**voom**	**vst**	**baySeq**	**EBSeq**	**SAMseq**
ShrinkSeq	**3259**	583	1125	985	1075	971	1049	192	803	1821
DESeq	583	**598**	598	567	588	589	587	191	523	592
edgeR	1125	598	**1160**	877	886	942	1013	194	753	1099
NBPSeq	985	567	877	**1082**	695	753	797	194	612	924
TSPM	1075	588	886	695	**1161**	891	907	191	794	1014
voom	971	589	942	753	891	**1009**	971	194	752	991
vst	1049	587	1013	797	907	971	**1095**	194	752	1061
baySeq	192	191	194	194	191	194	194	**195**	175	194
EBSeq	803	523	753	612	794	752	752	175	**819**	801
SAMseq	1821	592	1099	924	1014	991	1061	194	801	**1860**

The table contains the number of differentially expressed genes that are shared between each pair of methods, for the Bottomly data set (compare to Figure 5). The numbers on the diagonal, indicating the number of differentially expressed genes found by the respective methods, are highlighted in bold.

In Additional file 1: Figures S24-S28, we show the normalized counts (normalized using the normalization factors provided by the TMM method [8] together with the library sizes) across all samples for some of the genes found to be DE by only a single method. DESeq, edgeR, voom+limma, baySeq and EBSeq did not find any unique DE genes and hence there are no figures corresponding to these methods. From Additional file 1: Figures S24-S28, we noted that the DE genes found uniquely by ShrinkSeq, and to some extent for those found uniquely by SAMseq, tended to be reasonably highly expressed and consistently expressed across the samples from both conditions while for many of the other methods, the unique DE genes exhibited highly inconsistent counts even within conditions. The two genes found exclusively by vst+limma both had very low counts in all samples, as was the case for most genes found uniquely by TSPM.

In Additional file 1: Figure S29 we compare the gene ranking scores obtained by the different methods for the Bottomly data set (the scores were computed as described previously, recall that high scores correspond to genes considered DE). From this figure, we noted that edgeR, DESeq, voom+limma, vst+limma, TSPM and SAMseq tended to rank the genes similarly, while the rankings obtained by NBPSeq were less similar to these. The rankings obtained by baySeq and EBSeq were considerably different from the other rankings.

To further evaluate the performance of the methods, we applied them to the data set consisting of only the mice from the C57BL/6J strain, within which we defined two arbitrary sample classes of 5 samples each. The analysis was repeated five times for different arbitrary divisions. Under these conditions, we expect that no genes are truly DE. Nevertheless, most methods found differentially expressed genes in at least one instance. TSPM found by far the largest number of DE genes (Figure 5D), which supports our previous observation that this method may be too liberal. By studying the genes called DE in the five instances, we noted that the DE genes found by edgeR often overlapped with the DE genes found by NBPSeq, while only few of the DE genes called by TSPM overlapped with those found by the other methods. Also EBSeq tended to call unique genes, that were not found by any of the other methods. The lack of consensus among the DE genes found by the different methods may be a further indication that they are indeed false positives, and that the different methods tend to favor different types of patterns.

## Conclusions

In this paper, we have evaluated and compared eleven methods for differential expression analysis of RNA-seq data. Table 2 summarizes the main findings and observations. No single method among those evaluated here is optimal under all circumstances, and hence the method of choice in a particular situation depends on the experimental conditions. Among the methods evaluated in this paper, those based on a variance-stabilizing transformation combined with limma (i.e., voom+limma and vst+limma) performed well under many conditions, were relatively unaffected by outliers and were computationally fast, but they required at least 3 samples per condition to have sufficient power to detect any differentially expressed genes. As shown in the supplementary material (Additional file 1), they also performed worse when the dispersion differed between the two conditions. The non-parametric SAMseq, which was among the top performing methods for data sets with large sample sizes, required at least 4-5 samples per condition to have sufficient power to find DE genes. For highly expressed genes, the fold change required for statistical significance by SAMseq was lower than for many other methods, which can potentially compromise the biological significance of some of the statistically significantly DE genes. The same was true for ShrinkSeq, which however has an option for imposing a fold change requirement in the inference procedure.

**Table 2:** Summary of the main observations

	
DESeq	- Conservative with default settings. Becomes more conservative when outliers are introduced.
	- Generally low TPR.
	- Poor FDR control with 2 samples/condition, good FDR control for larger sample sizes, also with outliers.
	- Medium computational time requirement, increases slightly with sample size.
edgeR	- Slightly liberal for small sample sizes with default settings. Becomes more liberal when outliers are introduced.
	- Generally high TPR.
	- Poor FDR control in many cases, worse with outliers.
	- Medium computational time requirement, largely independent of sample size.
NBPSeq	- Liberal for all sample sizes. Becomes more liberal when outliers are introduced.
	- Medium TPR.
	- Poor FDR control, worse with outliers. Often truly non-DE genes are among those with smallest p-values.
	- Medium computational time requirement, increases slightly with sample size.
TSPM	- Overall highly sample-size dependent performance.
	- Liberal for small sample sizes, largely unaffected by outliers.
	- Very poor FDR control for small sample sizes, improves rapidly with increasing sample size. Largely unaffected by outliers.
	- When all genes are overdispersed, many truly non-DE genes are among the ones with smallest p-values. Remedied when the counts for some genes are Poisson distributed.
	- Medium computational time requirement, largely independent of sample size.
voom / vst	- Good type I error control, becomes more conservative when outliers are introduced.
	- Low power for small sample sizes. Medium TPR for larger sample sizes.
	- Good FDR control except for simulation study $B_{0}^{4000}$. Largely unaffected by introduction of outliers.
	- Computationally fast.
baySeq	- Highly variable results when all DE genes are regulated in the same direction. Less variability when the DE genes are regulated in different directions.
	- Low TPR. Largely unaffected by outliers.
	- Poor FDR control with 2 samples/condition, good for larger sample sizes in the absence of outliers. Poor FDR control in the presence of outliers.
	- Computationally slow, but allows parallelization.
EBSeq	- TPR relatively independent of sample size and presence of outliers.
	- Poor FDR control in most situations, relatively unaffected by outliers.
	- Medium computational time requirement, increases slightly with sample size.
NOISeq	- Not clear how to set the threshold for *q*_*NOISeq*_ to correspond to a given FDR threshold.
	- Performs well, in terms of false discovery curves, when the dispersion is different between the conditions (see supplementary material).
	- Computational time requirement highly dependent on sample size.
SAMseq	- Low power for small sample sizes. High TPR for large enough sample sizes.
	- Performs well also for simulation study $B_{0}^{4000}$.
	- Largely unaffected by introduction of outliers.
	- Computational time requirement highly dependent on sample size.
ShrinkSeq	- Often poor FDR control, but allows the user to use also a fold change threshold in the inference procedure.
	- High TPR.
	- Computationally slow, but allows parallelization.

The table summarizes the present study by means of the main observations and characteristic features for each of the evaluted methods. We have grouped voom+limma and vst+limma together since they performed overall very similarly.

Small sample sizes (2 samples per condition) imposed problems also for the methods that were indeed able to find differentially expressed genes, there leading to false discovery rates sometimes widely exceeding the desired threshold implied by the FDR cutoff. For the parametric methods this may be due to inaccuracies in the estimation of the mean and dispersion parameters. In our study, TSPM stood out as the method being most affected by the sample size, potentially due to the use of asymptotic statistics. Even though the development goes towards large sample sizes, and barcoding and multiplexing create opportunities to analyze more samples at a fixed cost, as of today RNA-seq experiments are often too expensive to allow extensive replication. The results conveyed in this study strongly suggest that the differentially expressed genes found between small collections of samples need to be interpreted with caution and that the true FDR may be several times higher than the selected FDR threshold.

DESeq, edgeR and NBPSeq are based on similar principles and showed, overall, relatively similar accuracy with respect to gene ranking. However, the sets of significantly differentially expressed genes at a pre-specified FDR threshold varied considerably between the methods, due to the different ways of estimating the dispersion parameters. With default settings and for reasonably large sample sizes, DESeq was often overly conservative, while edgeR and in particular NBPSeq often were too liberal and called a larger number of false (and true) DE genes. In the supplementary material (Additional file 1) we show that varying the parameters of edgeR and DESeq can have large effects on the results of the differential expression analysis, both in terms of the ability to control type I error rates and false discovery rates and in terms of the ability to detect the truly DE genes. These results also show that the recommended parameters (that are used in the main paper) are indeed well chosen and often provide the best results.

EBSeq, baySeq and ShrinkSeq use a different inferential approach, and estimate the posterior probability of being differentially expressed, for each gene. baySeq performed well under some conditions but the results were highly variable, especially when all DE genes were upregulated in one condition compared to the other. In the presence of outliers, EBSeq found a lower fraction of false positives than baySeq for large sample sizes, while the opposite was true for small sample sizes.

## Methods

In the following section we give a brief overview of the eleven methods for differential expression analysis that are evaluated and compared in the present paper. For more elaborate descriptions we refer to the original publications. All methods take their starting point in a count matrix, containing the number of reads mapping to each gene in each of the samples in the experiment. Nine of the methods work directly on the count data, while the remaining two transform the counts and feed the transformed values into the R package limma [10], which was originally developed for differential expression analysis of microarray data.

The methods working directly on the count data can be broadly divided into parametric (baySeq [14], EBSeq[24], ShrinkSeq [27], edgeR [23], DESeq [7], NBPSeq [15] and TSPM [13]) and non-parametric methods (NOISeq [25] and SAMseq [26]). The two-stage Poisson model (TSPM) proposed in [13] is based on a Poisson model for the counts, which is extended via a quasi-likelihood approach to allow for overdispersion if there is enough evidence for it in the data. Hence, the first step is to test each gene individually for evidence of overdispersion, in order to decide which of the two models to use for the differential expression analysis. The tests for differential expression are based on asymptotic statistics, which implies that the total count for each gene, across all samples, must not be too small. The authors therefore recommend that genes with a total count less than 10 are removed from the analysis. They also note that for the TSPM to work well, it may be important that there are indeed some genes for which there is no overdispersion.

Most of the remaining parametric models (baySeq, DESeq, EBSeq, edgeR and NBPSeq) use instead a Negative Binomial (NB) model to account for the overdispersion, while ShrinkSeq allows the user to select among a number of different distributions, including the NB and a zero-inflated NB distribution. DESeq, edgeR and NBPSeq take a classical hypothesis testing approach, while baySeq, EBSeq and ShrinkSeq instead are cast within a Bayesian framework. It is acknowledged that a crucial part of the inference procedure is to obtain a reliable estimate of the dispersion parameter for each gene, and hence considerable effort is put into this estimation. Due to the small sample size in most RNA-seq experiments it is difficult to estimate the gene-wise dispersion parameters reliably, which motivates information sharing across all genes in the data set in order to obtain more accurate estimates. Both DESeq, edgeR and NBPSeq incorporate information sharing in the dispersion estimation, and the way that this information sharing is done accounts for the main difference between the three methods. The first suggestion [12] was to assume that all genes had the same dispersion parameter. This could then be estimated from all the available data using a conditional maximum likelihood approach. A common dispersion for all genes may however be a too restrictive assumption, and so this procedure was developed further to allow for gene-wise dispersion estimates, but where the individual estimates were squeezed towards the common one using a weighted likelihood approach [31]. This method is used by edgeR. In contrast, DESeq and NBPSeq obtain the dispersion estimates by modeling the observed mean-variance (or the mean-dispersion) relationship for the genes in the data set using either parametric or local regression. After having obtained the fitted values, DESeq takes a conservative approach by defining the dispersion of a gene as the largest of the value obtained from the fitting and the individual dispersion estimate for the gene. NBPSeq does not take the same type of conservative approach as DESeq, and uses the fitted dispersion values only. After obtaining an estimate of the mean and the dispersion parameter for each gene, edgeR, DESeq and NBPSeq test for significant differential expression using either a variant of an exact test (for two-group comparisons) or a generalized linear model (allowing more complex experimental designs).

The approach used by baySeq and EBSeq is similar to the three previously mentioned methods in terms of the underlying NB model, but differs in terms of the inference procedure. For baySeq, the user defines a collection of *models*, each of which is essentially a partitioning of the samples into groups, where samples in the same group are assumed to share the same parameters of the underlying distribution. Within an empirical Bayes framework, baySeq then estimates the posterior probability of each model for each of the genes in the data set. Information from the entire set of genes is used to form an empirical prior distribution for the parameters in the NB model. EBSeq uses a similar approach, but assumes a parametric form of the prior distribution of the parameters, with hyperparameters that are shared between all the genes and estimated from the data.

ShrinkSeq, which also takes a Bayesian perspective, supports a number of different count models, including the NB and a zero-inflated NB. It provides shrinkage of the dispersion parameter, but also of other parameters such as the regression coefficients that are of interest for the inference. Furthermore, it incorporates a step for refining the priors, and subsequently the posteriors, non-parametrically after fitting the model for each feature.

The two non-parametric methods evaluated here, NOISeq and SAMseq, do not assume any particular distribution for the data. SAMseq is based on a Wilcoxon statistic, averaged over several resamplings of the data, and uses a sample permutation strategy to estimate a false discovery rate for different cutoff values for this statistic. These estimates are then used to define a q-value for each gene. NOISeq explores the distribution of fold-changes and absolute expression differences between the two contrasted conditions for the observed data, and compares this distribution to the corresponding distribution obtained by comparing pairs of samples belonging to the same condition (this is called the “noise distribution”). Briefly, NOISeq computes, for each gene, a statistic (here denoted *q*_*NOISeq*_) defined as the fraction of points from the noise distribution that correspond to a lower fold change and a lower absolute expression difference than those of the gene of interest in the original data.

Finally, the two transformation approaches (the variance stabilizing transformation provided in the DESeq R package and the voom transformation from the limma R package) aim to find a transformation of the counts to make them more amenable to analysis by traditional methods developed for differential expression analysis in the microarray context. The variance-stabilizing transformation provided in the DESeq R package (here denoted ‘vst’) explicitly computes the transformation by assuming a NB distribution and using dispersion estimates obtained as for DESeq. The ‘voom’ transformation from the limma R package essentially log-transforms the normalized counts and uses the mean-variance relationship for the transformed data to compute gene weights, which are then used by limma during the differential expression analysis.

In the present study, we focus on two-group comparisons only, since this is arguably the most common situation in practice. However, most of the evaluated methods support also more complex experimental designs. Most methods (edgeR, DESeq, NBPSeq, TSPM) achieve this through a generalized linear model (GLM) framework, where the user can specify desired contrasts to test. The limma package offers similarly flexible design options for the transformed data. The Bayesian methods (baySeq and EBSeq) allow the user to provide models defining collections of samples that are supposed to share the same distributional parameters, and return the posterior likelihood of each model thus defined. ShrinkSeq is based on the general framework of Gaussian latent models through the INLA approach [32], which allows very flexible experimental designs, including also random effects. It is also possible to impose a fold change threshold in the estimation of the posterior probabilities of differential expression. SAMseq provides nonparametric tests for various situations, such as paired and unpaired two-group comparisons, multigroup comparisons and survival analysis. NOISeq, in its current implementation, allows only two-group comparisons.

### Parameter choices

Many of the methods that are compared in this paper allow the user to select the value of certain parameters, that can affect the results in various ways. We have mostly used the default values provided in the implementations, but in the supplementary material (Additional file 1) we also provide some comparisons of the performances for different choices of the parameter values. This section summarizes the parameter values that were used for the evaluations in the main paper. For more detailed information about the meaning of the different parameters, we refer to the original publications describing the respective methods.

For edgeR, we used the TMM method (Trimmed Mean of M-values [8]) to calculate normalization factors between samples. We used tagwise dispersion estimates, squeezed towards a trended estimate computed by the ‘moving average’ approach. We performed an exact test to find genes that were differentially expressed between two conditions.

For DESeq, we computed a pooled estimate of the dispersion parameter for each gene. We used local regression to find the mean-variance relationship and employed the conservative approach of selecting the largest among the fitted value and the individual dispersion estimate for each gene. Also here, we used the implemented exact test to find DE genes. The local regression approach was also used in the variance-stabilizing transformation provided by the DESeq package (denoted ‘vst’). Here, we used instead the ‘blind’ option for the dispersion estimation.

Also for TSPM, baySeq, voom and NBPSeq we used the TMM method to compute normalization factors. For NOISeq, we normalized the counts using the TMM method before feeding the data into the differential expression analysis. Furthermore, for NBPSeq we used the ‘NBP’ parametrization of the Negative Binomial distribution. For baySeq, we assumed a Negative Binomial distribution and used the quasi-likelihood approach to estimate priors. We used a sample size of 5,000 to estimate the priors. Furthermore, we assumed equal dispersion for a gene in the two sample groups and used the ‘BIC’ option for the prior re-estimation step. For EBSeq, we used the default ‘median’ normalization method, that is, the normalization provided with DESeq [7].

Before applying ShrinkSeq, we normalized the counts using TMM normalization factors. Within ShrinkSeq we then employed a zero-inflated Negative Binomial distribution, and applied shrinkage to the dispersion parameter as well as the regression coefficient of interest in the inference procedure. To make the results from ShrinkSeq comparable to those from the other methods, we did not impose a non-zero fold change threshold when estimating the false discovery rates.

### Data sets

Most of the evaluations in this paper are based on synthetic data, where we could control the settings and the true differential expression status of each gene. We generated the counts for each gene from a Negative Binomial distribution, with mean and dispersion parameters estimated from real RNA-seq data, following the same approach as in [20]. We refer to the supplementary material (Additional file 1) for more detailed information about how the parameters were estimated. All methods were run on the same data sets.

We let *G* = {*g*_1_, …, *g*_|*G*|_} denote the set of genes in our data set. In the synthetic data sets, we took |G|=12,500. Similarly, we let *S* = {*s*_1_, …, *s*_|*S*|_} denote the set of samples, and assumed that these were partitioned into two subsets S_1_ and S_2_. In our experiments, we let |S_1_|=|S_2_| and we thought of S_1_ as the “control” group of samples and S_2_ as a group of samples with an abnormal phenotype. We let $G_{\mathrm{DE}}^{\mathrm{up}}\subseteq G$ denote the set of genes that were differentially expressed between the two sample groups, and which were upregulated in S_2_. Similarly, $G_{\mathrm{DE}}^{\mathrm{down}}\subseteq G$ denoted the set of genes that were downregulated in S_2_ compared to S_1_.

The random variable representing the count for gene g in sample s was denoted *Y*_*gs*_. It was modeled by a Negative Binomial distribution, following the approach outlined in [8], by letting

$$Y_{\mathrm{gs}}\sim \mathrm{NB}\left(\mathrm{mean}=\mu_{\mathrm{gs}},\mathrm{var}=\mu_{\mathrm{gs}}\left(1+\mu_{\mathrm{gs}}\phi_{\mathrm{gs}}\right)\right).$$

Here, *ϕ*_*gs*_ is the dispersion parameter, controlling the level of overdispersion. Moreover,

$$\;\mu_{\mathrm{gs}}=E\left[Y_{\mathrm{gs}}\right]=\frac{\lambda_{\mathrm{gc}\left(s\right)}}{\sum_{g\in G}\lambda_{\mathrm{gc}\left(s\right)}}M_{s}$$

where *M*_*s*_ is the sequencing depth for sample s, which we defined as *M*_*s*_ = 10^7^*U*_*s*_ for *U*_*s*_ ~ *Unif* [0.7, 1.4], and *c*(*s*) ∈ {*S*_1_, *S*_2_} denoted the condition for sample s. We let the dispersion parameter *φ*_gs_ be the same in the two sample groups, that is, *φ*_gs_ = *φ*_g_ for all *s*.

For each gene, we drew a pair of values $\lambda_{gS_{1}}$ and *φ*_g_ from those estimated from the real RNA-seq data. We then defined $\lambda_{gS_{2}}=\gamma_{g}^{v_{g}}\lambda_{gS_{1}}$ where $\gamma_{g}=w_{g}+{\bar{\gamma }}_{g},$${\bar{\gamma }}_{g}\sim \mathrm{Exp}\left(1\right)$ and

$$v_{g}=\{\begin{array}{c} 1\;\mathrm{if}\;g\in G_{\mathrm{DE}}^{\mathrm{up}}\\ -1\;\mathrm{if}\;g\in G_{\mathrm{DE}}^{\mathrm{down}}\\ 0\;\mathrm{otherwise} \end{array}$$

The parameter *w*_*g*_ denoted the lower bound on the differential expression between the two groups. In our simulations, we let *w*_*g*_ = 1.5 for all g.

To simulate different real situations, we also evaluated the effect of generating the counts for half of the genes using a Poisson distribution (i.e., without overdispersion, simulation studies denoted ‘*P*’). Furthermore, we studied the effect of including outliers with extremely high counts. The outliers were introduced in two different ways. For the ‘single’ outlier simulation studies (denoted ‘*S*’), we selected 10% of the genes, and for each of these genes we selected a single sample for which we multiplied the observed count with a randomly selected factor between 5 and 10. For the ‘random’ outlier simulation studies (denoted ‘*R*’), we considered each observed count independently, and with probability 0.05 we multiplied a count by a randomly selected factor between 5 and 10. Table 3 summarizes the parameter values that were used in the different simulation studies. For each synthetic data set, we filtered out all genes for which the total count across all samples was less than 10 before the differential expression analysis was performed.

**Table 3:** Summary of the parameters used to generate the synthetic data sets

**Sim. study**	$|G_{\mathrm{DE}}^{\mathrm{up}}|$	$|G_{\mathrm{DE}}^{\mathrm{down}}|$	**|{*g;*** ***ϕ***_***g***_** = 0}|**	**‘Single’ outlier fraction**	**‘Random’ outlier fraction**
$B_{0}^{0}$	0	0	0	0	0
$B_{0}^{1250}$	1,250	0	0	0	0
$B_{625}^{625}$	625	625	0	0	0
$B_{0}^{4000}$	4,000	0	0	0	0
$B_{2000}^{2000}$	2,000	2,000	0	0	0
$P_{0}^{0}$	0	0	6,250	0	0
$P_{625}^{625}$	625	625	6,250	0	0
$S_{0}^{0}$	0	0	0	10%	0
$S_{625}^{625}$	625	625	0	10%	0
$R_{0}^{0}$	0	0	0	0	5%
$R_{625}^{625}$	625	625	0	0	5%

In all synthetic data sets, the observations were distributed between two conditions (denoted S_1_ and S_2_), with the same number of observations (2, 5 or 10) in each condition. We let $\left|G_{\mathrm{DE}}^{\mathrm{up}}\right|$ and $\left|G_{\mathrm{DE}}^{\mathrm{down}}\right|$ denote, respectively, the number of genes that were up- and downregulated in condition S_2_ compared to S_1_. The number of genes whose counts were drawn from a Poisson distribution (i.e., with the dispersion parameter equal to zero) is given by |{*g*; *ϕ*_*g*_ = 0}|. The ‘single’ outlier fraction denotes the fraction of the genes for which we selected a single sample and multiplied the corresponding count with a factor between 5 and 10. The ‘random’ outlier fraction denotes the fraction of counts that were selected randomly (among all counts) and multiplied with a factor between 5 and 10. The notation for the simulation studies (leftmost column) summarizes the type of simulation (*B* - ‘baseline’, *P* - ‘Poisson’, *S* - ‘single outlier’, *R* - ‘random outlier‘), the number of DE genes that are upregulated in S_2_ (i.e., $\left|G_{\mathrm{DE}}^{\mathrm{up}}\right|$, in the superscript) and the number of DE genes that are downregulated in S_2_ (i.e., $|G_{\mathrm{DE}}^{\mathrm{down}}|,$, in the subscript).

In addition to the synthetic data, we also considered a real RNA-seq data set [30] that we downloaded from http://bowtie-bio.sourceforge.net/recount/. The data set contained RNA-seq data taken from 21 samples from two different mouse strains. Also for this data set we filtered out all genes for which the total count across the 21 samples did not exceed 10, which left 11,870 genes in the data set. In the supplementary material, we analyse two other real data sets [33,34], downloaded from the same source.

## Competing interest

The authors declare that they have no competing interests.

## Authors’ contributions

CS and MD contributed to the design of the study, the interpretation of the results and the writing of the manuscript. CS performed the implementation and the numerical experiments. Both authors read and approved the final manuscript.

## Supplementary Material

Additional file 1 **Contains supplementary figures referred to in the text.** Here, we also evaluate the effect of selecting different values for the parameters of edgeR and DESeq and evaluate two additional transformation-based methods, and we evaluate the effect of simulating data with different dispersion parameter in the two compared conditions. We also present some comparisons based on data sets with 3 samples per condition. The file also contains information regarding the estimation of the mean and dispersion parameters from real data, and an additional analysis of two real RNA-seq data sets. Finally, it contains sample R code to run the differential expression analysis and estimates of the computational time requirements for the different methods. Click here for file
