# Supplementary material for: A comparison of methods for differential expression analysis of RNA-seq data
Source: BMC Bioinformatics. 2013 Mar 9;14:91. doi: 10.1186/1471-2105-14-91 (PMC3608160; doi:10.1186/1471-2105-14-91)
Supplement: Additional file 1 — Contains supplementary figures referred to in the text. Here, we also evaluate the effect of selecting different values for the parameters of edgeR and DESeq and evaluate two additional transformation-based methods, and we evaluate the effect of simulating data with different dispersion parameter in the two compared conditions. We also present some comparisons based on data sets with 3 samples per condition. The file also contains information regarding the estimation of the mean and dispersion parameters from real data, and an additional analysis of two real RNA-seq data sets. Finally, it contains sample R code to run the differential expression analysis and estimates of the computational time requirements for the different methods. [file 1471-2105-14-91-S1.pdf]

A comparison of methods for differential expression analysis of RNA-seq data – Supplementary Material

Charlotte Soneson and Mauro Delorenzi

Contents

1 Estimating mean and dispersion parameters from real data **2**

2 Effect of different parameter choices **5**
2.1 edgeR 5
2.2 DESeq 9
2.3 Transformations 11

3 Analysis of the Blekhman data set **13**

4 Analysis of the Hammer data set **14**

5 Simulations with 3 samples/condition **16**

6 Simulations with unequal dispersion across conditions **19**

7 R commands **24**
7.1 edgeR 24
7.2 DESeq 24
7.3 NBPSeq 24
7.4 baySeq 25
7.5 EBSeq 25
7.6 TSPM 26
7.7 SAMseq 26
7.8 NOISeq 26
7.9 voom+limma 27
7.10 vst+limma 27
7.11 ShrinkSeq 27

8 Computational time requirement **29**

9 Supplementary Figures **31**

1 Estimating mean and dispersion parameters from real data

The mean and dispersion parameters that are used in the simulation of the synthetic data sets are estimated from two real RNA-seq data sets available from the `tweeDEseqCountData` R package and from `http://bowtie-bio.sourceforge.net/recount/` [5], following the methodology outlined by [12]. The Pickrell data set [8] contains RNA-seq data from 69 unrelated Nigerian individuals. The Cheung data set [4] contains RNA-seq data from 41 unrelated Caucasian individuals of European descent. Each data set was processed individually according to the following description and in the end, the obtained mean and dispersion estimates from the two data sets were merged to form the final set of mean-dispersion pairs.

First, we removed all samples for which the library size was smaller than 2 million, and all genes for which the average count across the samples was less than 1. Then, we resampled the reads for each sample so that the library sizes for all samples were equal to the smallest library size. For each gene, we then found maximum likelihood estimates of the mean μ_g and the dispersion ϕ_g from the resampled counts. The log-likelihood function for N iid variables from a Negative Binomial distribution, given counts $y_1, \ldots, y_N$, is (as in [12])

$$\begin{aligned}\ell(\mu, \phi | y_1, \ldots, y_N) = &\sum_{j=1}^{N} \log \Gamma(y_j + 1/\phi) - N \log \Gamma(1/\phi) \\ &- \sum_{j=1}^{N} \log \Gamma(y_j + 1) + \sum_{j=1}^{N} y_j \log \left(\frac{\mu\phi}{1 + \mu\phi} \right) - \frac{N}{\phi} \log(1 + \mu\phi).\end{aligned}$$

The maximum likelihood estimate (MLE) of μ_g is first obtained as the average count for gene g across all samples. Then, we estimate ϕ_g by numerically maximizing the log-likelihood function using the observed values for the counts $y_1, \ldots, y_N$.

Supplementary Figure 1 shows the estimates of μ_g and ϕ_g obtained from the two data sets in different colors (most of the black dots, from the Pickrell data set, are hidden behind the red ones, from the Cheung data set). We notice an excellent agreement between the estimates from the two data sets, which justifies the merging. To generate the synthetic data sets we then, for each gene, sample a pair (μ_g, ϕ_g) from those obtained by the estimation described here.

To see how well some characteristics of the simulated data correspond to those of real data, we compare the mean-variance relationship obtained for the Pickrell data to that for normalized counts from one instance of simulation study B_0^0 (Supplementary Figure 2(a)). There is good agreement, suggesting that the generative data model fits well with reality. We also compute the average count for each gene, and compare the distributions of these between the real and synthetic data sets (Supplementary Figure 2(b)). Also here we note a good agreement between the data sets.

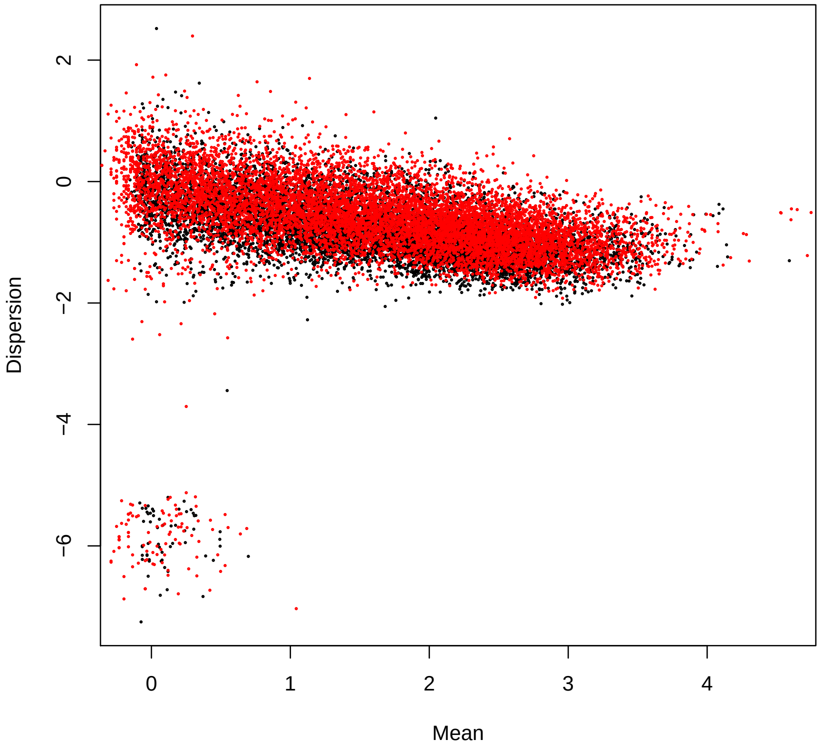

Supplementary Figure 1. *The MLEs of the mean and dispersion parameters based on the Pickrell data set (black dots) and the Cheung data set (red dots). The mean and dispersion parameters for the simulated data sets are sampled from these pairs of values.*

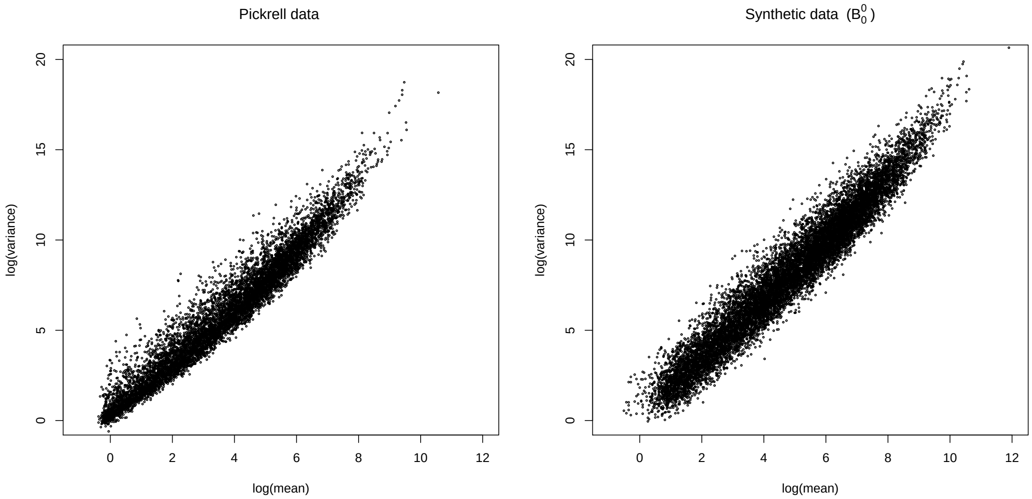

(a)

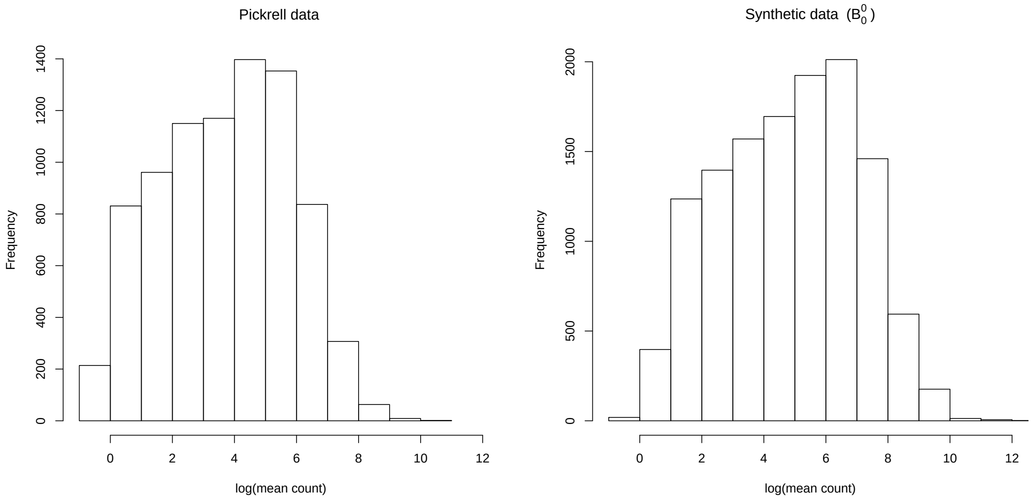

(b)

Supplementary Figure 2. *Comparison between real and synthetic data. (a) Mean-variance relationship. (b) Average count distribution.*

2 Effect of different parameter choices

In this section, we examine the effect of selecting different options for the input parameters for edgeR and DESeq, and we also evaluate two additional transformations that can be used to transform the counts before applying limma to find differentially expressed genes.

2.1 edgeR

In edgeR [9], the user is given the opportunity to select which method to use for normalization of counts between samples (the available methods are TMM [10], RLE [1] and upper quartile [3]). For two-group comparisons, the user can choose between performing an exact test [11] and using a generalized linear model (GLM) based on the NB distribution [7]. For more complex experimental designs, only the GLM option is available. Finally, the user can decide how to estimate the dispersion parameter for each gene. There are three main options, namely using a common dispersion estimate for all genes, computing a trended estimate (allowing the dispersion to depend on the mean), or estimating a gene-wise dispersion. The gene-wise dispersion estimate is squeezed towards either the common estimate or towards the trended estimate by means of a weighted likelihood procedure. Depending on the test that is used for the differential expression analysis, the dispersion estimation is performed in different ways. The so called qCML method [11] is used for the exact test, but it is not applicable for the more general GLM, where instead the Cox-Reid estimator is the default choice [7], although the 'Pearson and 'deviance' options are available as well.

In Supplementary Figure 3 we show the effect of choosing these parameters in different ways, for simulation studies B_0^0 and B_{625}^{625}, in terms of type I error rate (simulation study B_0^0), AUC and the true FDR and TPR when setting the significance threshold at a false discovery rate of 0.05 (simulation study B_{625}^{625}). Recall that for simulation study B_{625}^{625}, 1,250 genes are truly DE, and the set of DE genes consists of both genes upregulated in S_2 and genes downregulated in S_2 compared to S_1. The parameter combination corresponding to the one used in the main paper is the one denoted 'edgeR.exact.TMM.tagwise.movingave'. First, we note that for these simulation settings, the choice of normalization method appears to have little effect in all respects (compare the results for 'edgeR.exact.XXX.tagwise.movingave' with XXX replaced by TMM, RLE and upper, respectively). We made the same observations for most of the other simulation settings as well, except when all DE genes were upregulated in condition S_2 compared to S_1 (i.e., simulation studies B_0^{1250} and B_0^{4000}). In these situations, the upper quartile normalization gave slightly worse results than the TMM and RLE methods, in terms of higher FDR and lower TPR and AUC (Supplementary Figures 4(a)-4(c)).

In Supplementary Figure 3(b), we further note that all parameter choices have only minor effects on the ability to rank the truly DE genes before the truly non-DE ones (summarized by the AUC). This was observed for almost all simulation studies, and the largest deviation was found for simulation study B_0^{4000} (Supplementary Figure 4(a)). However, the sets of genes called DE are quite different for different parameter values. As can be seen in Supplementary Figures 3(a), 3(c) and 3(d), using a common dispersion estimate gives the highest number of false positive findings and generally the lowest number of true positive findings. Seen over all simulation studies, the common dispersion estimate gives the

highest FDR in all cases except for simulation setting B_0^{4000} (i.e., when almost one third of the genes are DE, and all are regulated in the same direction). Also, squeezing the tagwise estimates towards a trended estimate generally gives better results than squeezing towards a common estimate (the 'notrend' option).

For the GLM option, the choice of tagwise dispersion estimation procedure ('deviance' or 'CoxReid') has no detectable influence on the results in any of our simulation studies. Finally, from the results presented here it seems that the GLM approach finds somewhat more significant genes (both true and false) than the exact test.

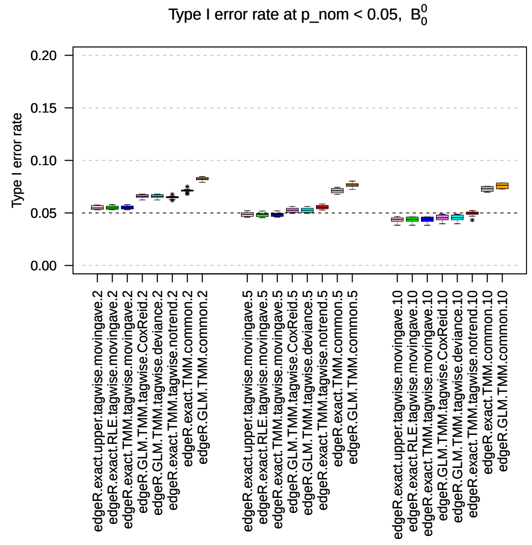

(a)

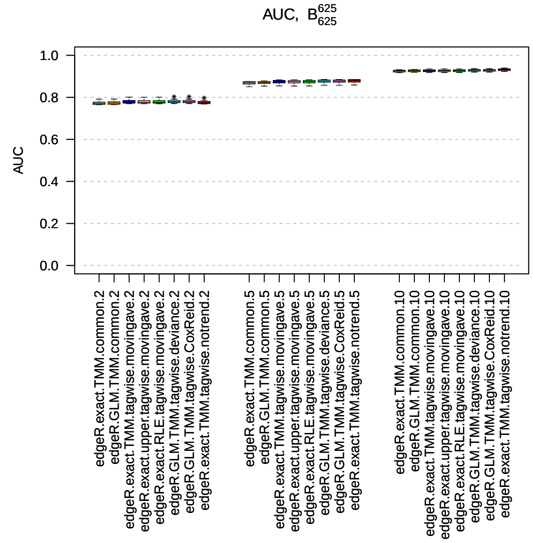

(b)

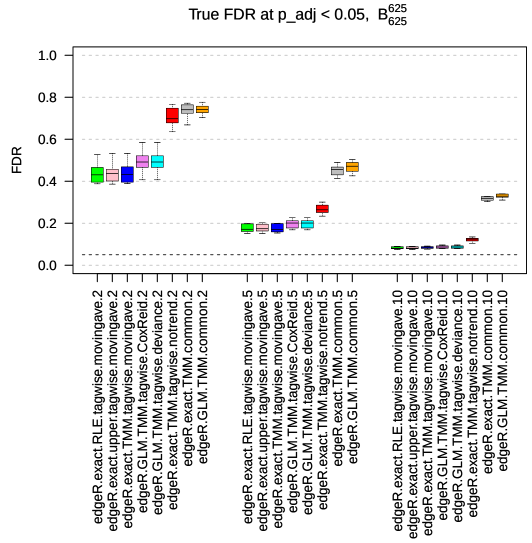

(c)

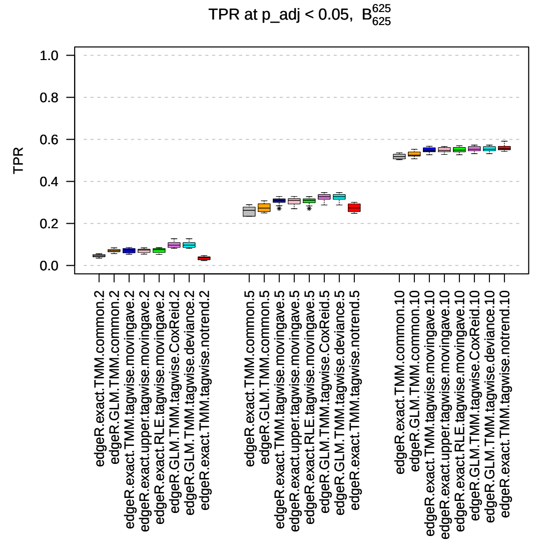

(d)

Supplementary Figure 3. *The effect of varying parameter values on the results obtained by edgeR, for simulation studies B_0^0 and B_{625}^{625}. (a) Observed type I error rate at a nominal p-value threshold of 0.05, for simulation study B_0^0. (b) Area under the ROC curve (AUC) for simulation study B_{625}^{625}. (c) True FDR for a significance threshold put at an adjusted p-value of 0.05, for simulation study B_{625}^{625}. (d) True positive rate for a significance threshold put at an adjusted p-value of 0.05, for simulation study B_{625}^{625}. The last digit of the tick labels signifies the number of samples per condition in the respective experiments (2, 5 or 10).*

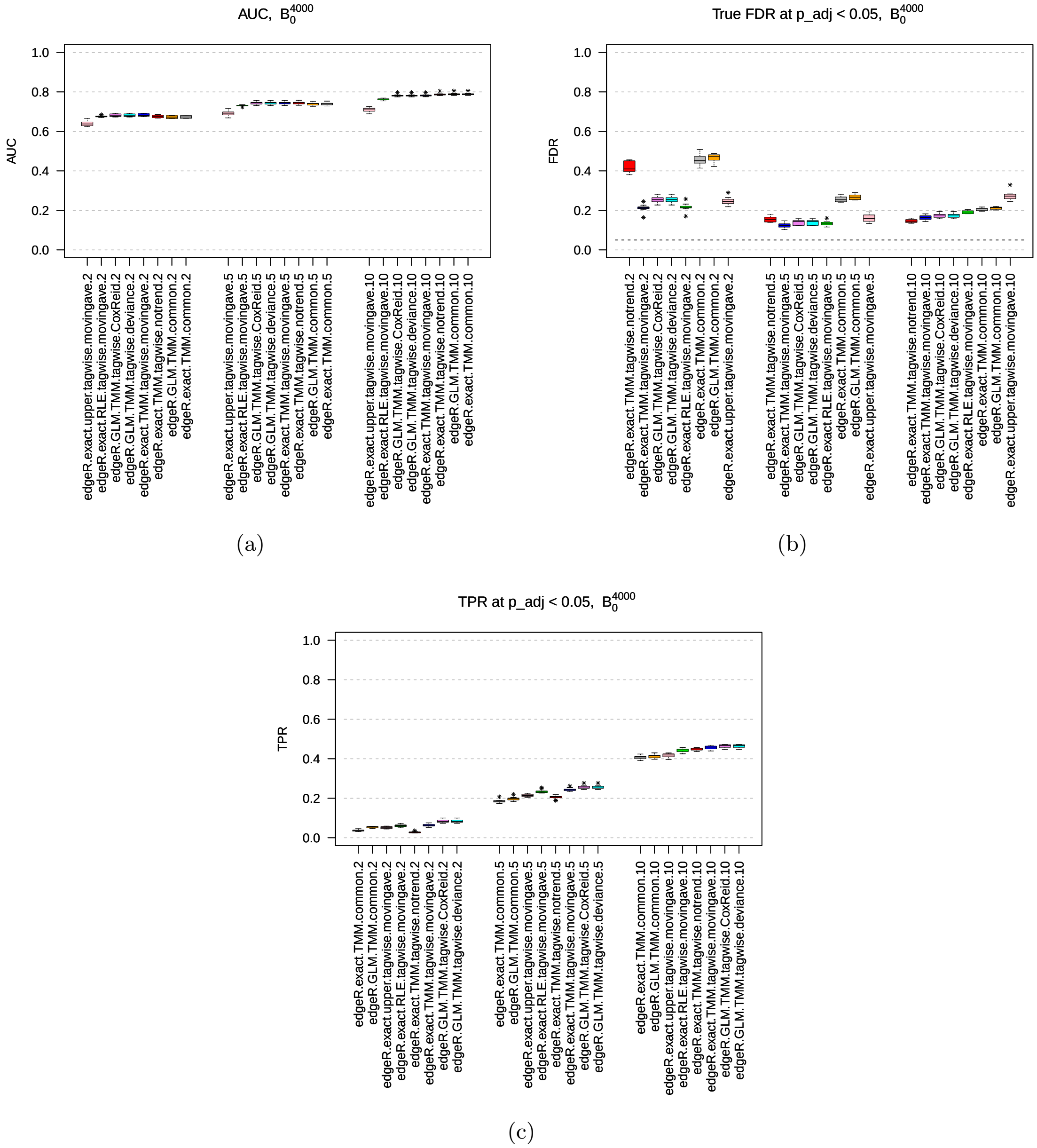

Supplementary Figure 4. *The effect of varying parameter values on the results obtained by edgeR, for simulation study B_0^{4000}. (a) Area under the ROC curve (AUC). (b) True FDR for a significance threshold put at an adjusted p-value of 0.05. (c) True positive rate for a significance threshold put at an adjusted p-value of 0.05.*

2.2 DESeq

With DESeq [1], just as for edgeR, the user can choose whether to perform an exact test (here referred to as 'nbinom') or to use a GLM. Also here, there are opportunities for selecting how the dispersion estimation is performed. As described in the main paper, the dispersion parameters are obtained from the observed mean-variance relationship for the genes in the data set. The user can choose whether to model this relationship parametrically or by means of local regression. It is also possible to choose if the dispersion should be estimated for each condition separately, if it should be estimated separately and then pooled to a common estimate, or if the samples from all conditions should be pooled before the estimation (the 'blind' option). After the mean-variance relationship is modeled, the user can also select if the final dispersion estimate for a gene should be the fitted value ('fit' below), the original estimate (regardless of the fitting, 'gene' below) or the largest of the two values ('max' below).

Supplementary Figure 5 shows the effect of selecting these parameters differently, in simulation studies B_0^0 and B_{625}^{625}. The combination used in the main paper is the one denoted 'DESeq.nbinom.pooled.max.local'. Clearly, as for edgeR, the parameter values have only a marginal influence on the ability to rank truly DE genes before truly non-DE genes (Supplementary Figure 5(b)). The same observation was made across all simulation studies. However, the set of genes called differentially expressed is highly affected by the different parameter choices. Overall, taking the conservative approach of selecting the largest of the individual dispersion estimate and the fitted value naturally gives the lowest numbers of differentially expressed genes (both true and false). The other choices (gene-wise or fitted values) are generally too liberal and not able to control the false discovery rate or the type I error satisfactorily for any simulation setting. As expected, the gene-wise estimates are very poor for the smallest sample size, since it is very difficult to estimate gene-wise dispersions accurately based on only few samples, without borrowing information across genes. The performance when using the gene-wise dispersion estimates improves considerably with increasing sample size.

The choice of parametric or local modeling of the mean-variance relationship appears to have only a minor impact on the results. As for edgeR, the GLM approach seems to give slightly more significantly differentially expressed genes (both true and false).

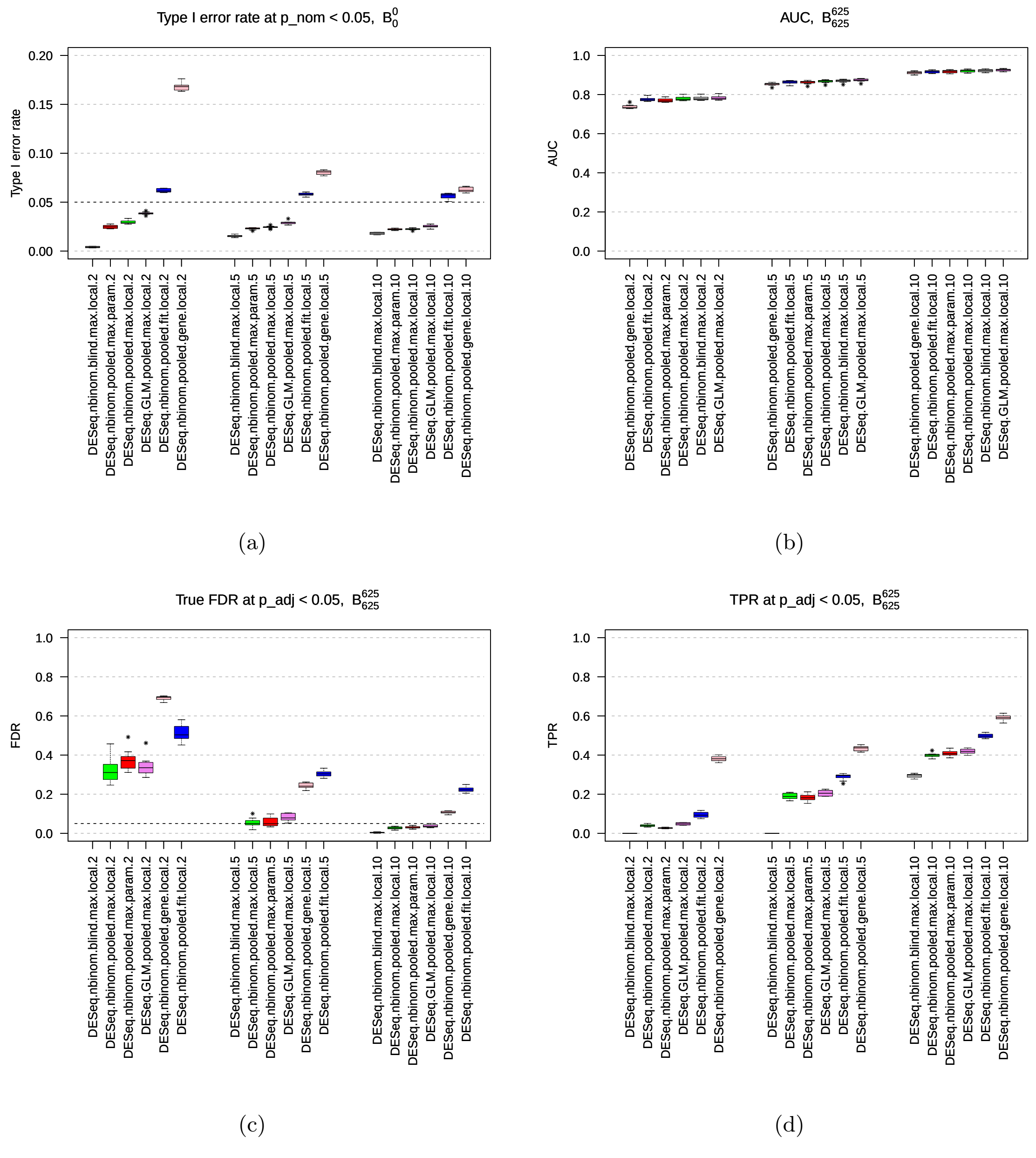

Supplementary Figure 5. *The effect of varying parameter values on the results obtained by DESeq, for simulation studies B_0^0 and B_{625}^{625}. (a) Observed type I error rate at a nominal p-value threshold of 0.05, for simulation study B_0^0. (b) Area under the ROC curve (AUC) for simulation study B_{625}^{625}. (c) True FDR for a significance threshold put at an adjusted p-value of 0.05, for simulation study B_{625}^{625}. (d) True positive rate for a significance threshold put at an adjusted p-value of 0.05, for simulation study B_{625}^{625}.*

2.3 Transformations

In the main paper, we studied two variance-stabilizing transformations that were specifically developed for RNA-seq data. Here, we compare the performance of these methods to two other transformations. First, we consider the log-transformation, more precisely we transform the count for gene g in sample s by

$$y_{gs} \mapsto \log\left(\frac{y_{gs} + 0.5}{nf_s \cdot M_s} \cdot 10^6\right) \quad (1)$$

where nf_s is the TMM normalization factor and M_s is the library size for sample s. This is the same transformation as is used by voom, but as we noted in the main article, voom proceeds by estimating gene weights from the mean-variance relationship for the transformed data, and uses these weights during the differential expression analysis with limma. We also consider replacing the log in equation (1) by a square root transformation.

Supplementary Figure 6 summarizes the results for the four different transformations, for simulation studies B_0^0 and B_{625}^{625}. Generally, the results are quite similar, the largest deviation being between the square root transformation and the other three, for small sample sizes. All transformations manage to control the FDR at least close to the desired level, but lacks power to detect differentially expressed genes for the smallest sample size. In the present example, the extra gene weight estimation provided by voom appears to provide the biggest advantage over only the log-transformation for the medium sample sizes, in terms of higher true positive rate and slightly lower false discovery rate. For large sample sizes, the performances of voom and the log-transformation were more similar. The same observation can be done in general for most simulation studies.

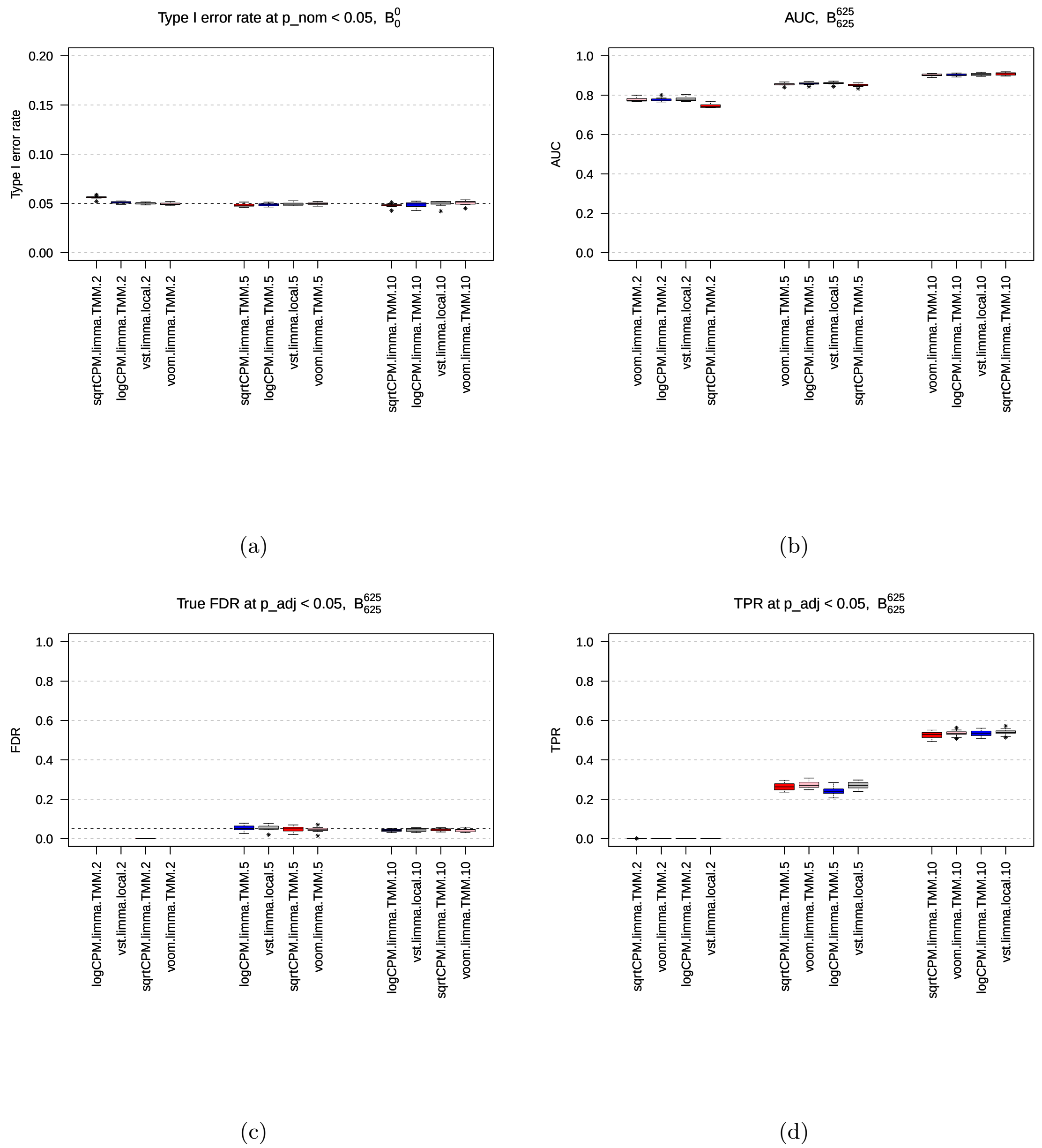

Supplementary Figure 6. *The effect of varying parameter values on the results obtained by different transformations, combined with limma, simulation studies* B_0^0 *and* B_{625}^{625}. *(a) Observed type I error rate at a nominal p-value threshold of 0.05, for simulation study* B_0^0. *(b) Area under the ROC curve (AUC) for simulation study* B_{625}^{625}. *(c) True FDR for a significance threshold put at an adjusted p-value of 0.05, for simulation study* B_{625}^{625}. *(d) True positive rate for a significance threshold put at an adjusted p-value of 0.05, for simulation study* B_{625}^{625}.

3 Analysis of the Blekhman data set

This data set [2] contains RNA-seq counts from liver samples from three men and three women. It was downloaded from `bowtie-bio.sourceforge.net/recount/`. After filtering out genes with less than 10 counts in total across all samples, the data set contains 8,031 genes. We applied the different methods, with the same parameter choices as in the main article, and recorded for each of them the set of DE genes found at an FDR threshold of 0.05, when contrasting the expression levels among men and women. Only four of the methods, namely TSPM, NBPSeq, EBSeq and edgeR, found any DE genes at all (Supplementary Figure 7). Also for these four methods, the number of DE genes at the imposed significance threshold was very low. Comparing the sets of DE genes, we noted that 122 of the 127 DE genes found by TSPM were not shared by any of the other methods. Similarly, 34 of the 48 DE genes found by EBSeq were unique to this method. On the other hand, the 15 DE genes found by edgeR formed a subset of the 57 DE genes found by NBPSeq. No gene was found to be DE by all four methods. Comparing to the within-group comparison for the Bottomly data set in the main article we note the same pattern in this data set, which suggests that the DE genes found by the four methods in the Blekhman data set may actually be false positives.

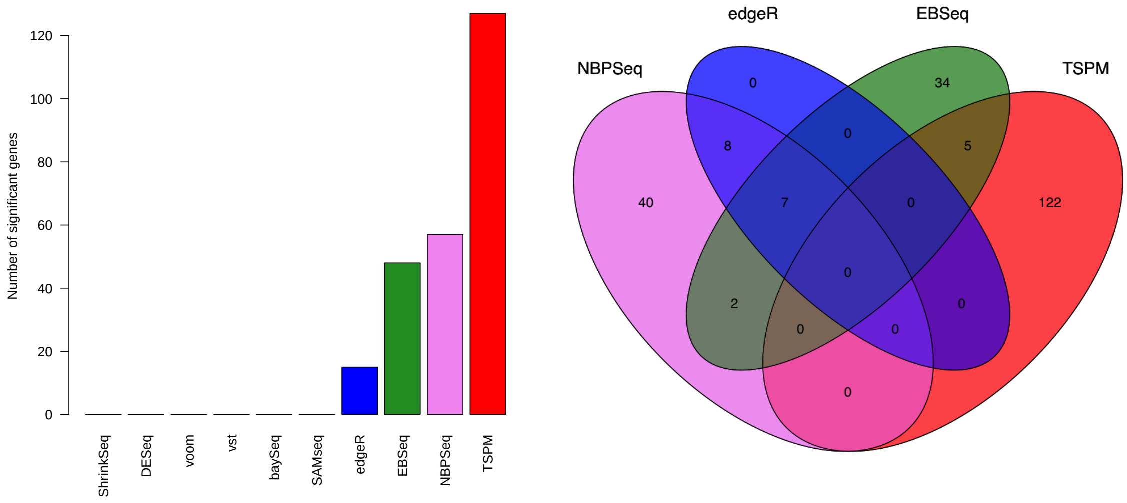

(a) (b)

Supplementary Figure 7. *The number of DE genes found by the different methods for the Blekhman data set (panel (a)) and the overlap between the sets of DE genes found by the methods (panel (b)).*

4 Analysis of the Hammer data set

This data set [6] contains RNA-seq counts from four rats with chronic neuropathic pain and four control rats. It was downloaded from `bowtie-bio.sourceforge.net/recount/`. After filtering out genes with less than 10 counts in total across all samples, the data set contains 14,228 genes. As above, we applied the different methods and recorded the set of genes that were found to be differentially expressed between controls and rats with chronic neuropathic pain for each of the methods. The results are shown in Supplementary Figure 8. Supplementary Figure 8(a) shows that ShrinkSeq and SAMseq found the highest number of DE genes, while baySeq and EBSeq found the smallest numbers. Supplementary Figures 8(b)-8(d) show the overlap between the sets of DE genes found by the different methods, for four methods at a time to increase the interpretability. Supplementary Figure 8(b) compares four of the methods that are based on a NB model (edgeR, DESeq, NBPSeq and baySeq). Among these methods, the sets of DE genes formed almost a nested sequence, where most of the DE genes found by a method returning a lower number of DE genes were found also by the methods returning more DE genes. Supplementary Figure 8(c) compares four of the remaining methods. We did not see the same nested structure for these methods, mainly since TSPM found a set of DE genes that were not shared by any of the other methods. Finally, Supplementary Figure 8(d) compares baySeq and EBSeq to the two transformation-based methods. Supplementary Table 1 shows the overlap between the sets of called DE genes for each pair of methods.

Supplementary Table 1. *The overlap between the sets of called DE genes for each pair of methods in the Hammer data set. The diagonal elements, indicating the number of DE genes found by each method, are highlighted in bold.*

	ShrinkSeq	DESeq	edgeR	NBPSeq	TSPM	voom	vst	baySeq	EBSeq	SAMseq
ShrinkSeq	**10361**	6134	6873	7453	6064	6952	5792	4491	4131	9183
DESeq	6134	**6153**	6149	6121	4689	6150	5373	4446	3371	6153
edgeR	6873	6149	**6895**	6811	5072	6825	5534	4500	3797	6890
NBPSeq	7453	6121	6811	**7478**	5409	6874	5594	4499	3937	7392
TSPM	6064	4689	5072	5409	**6118**	5087	4402	3355	2143	6095
voom	6952	6150	6825	6874	5087	**6982**	5506	4501	3843	6955
vst	5792	5373	5534	5594	4402	5506	**5817**	4445	3105	5803
baySeq	4491	4446	4500	4499	3355	4501	4445	**4501**	2808	4500
EBSeq	4131	3371	3797	3937	2143	3843	3105	2808	**4164**	4039
SAMseq	9183	6153	6890	7392	6095	6955	5803	4500	4039	**9403**

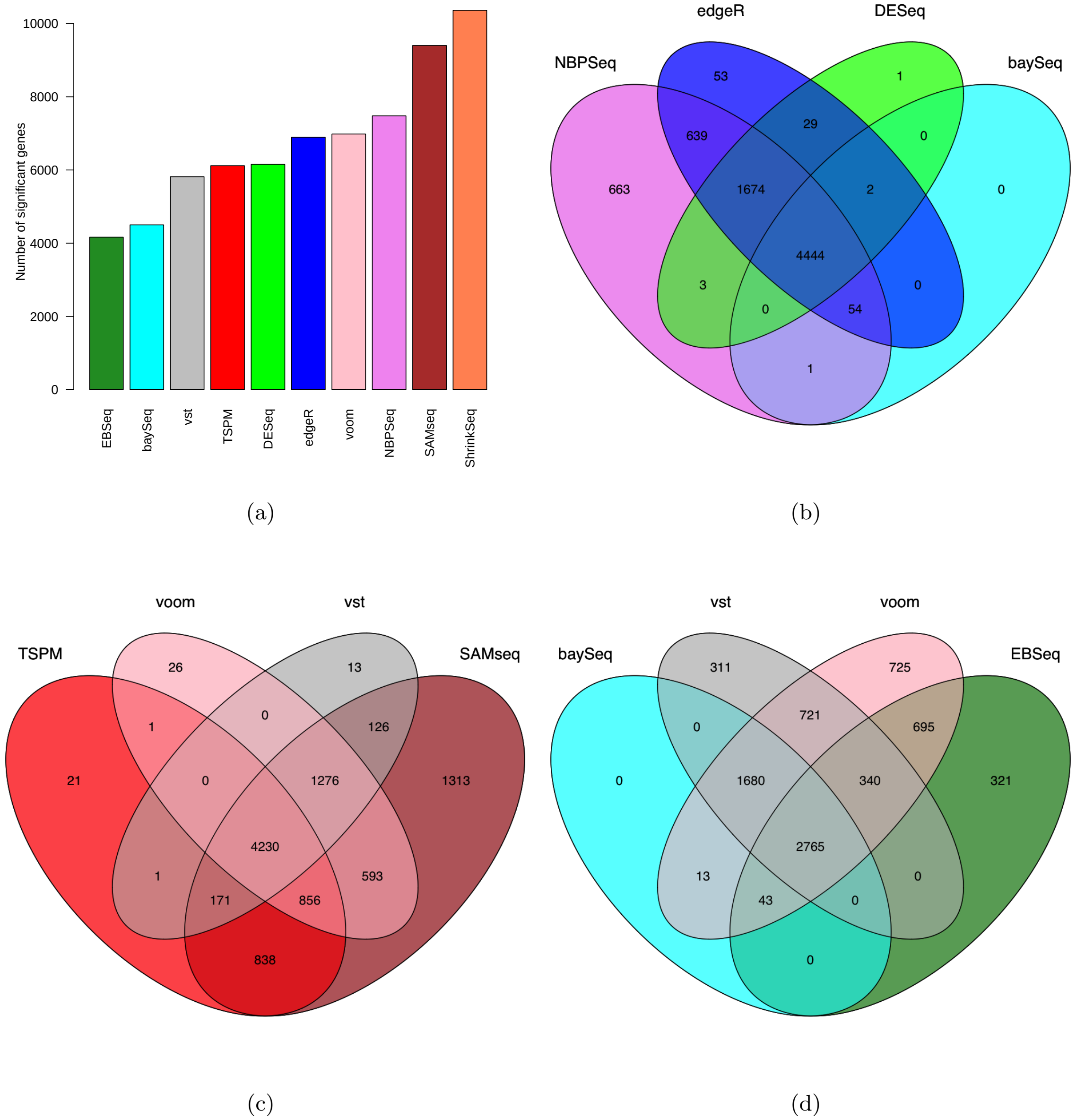

Supplementary Figure 8. *(a) The number of DE genes found by the different methods for the Hammer data set. (b) - (d) Overlaps among the sets of DE genes found by different subsets of the compared methods.*

5 Simulations with 3 samples/condition

In the main article, we present results for simulated data with 2, 5, and 10 samples per condition. These values were chosen to represent a large variety of different experiments and to compare the methods under different settings. However, in real RNA-seq experiments the choice is often still between having two or three replicates per condition, and for this reason we show here some comparisons of the methods also for 3 samples per condition, and relate them to the results for 2 and 5 samples per condition.

In Figure 9 we show the type I error rate for simulation study B_0^0, at a nominal p-value threshold of 0.05. The TSPM method is most clearly affected by changing sample size, with a noticeable drop in error rate between 2 and 3 samples per condition.

Figure 10 shows the AUC (top left panel), representative false discovery curves (top right panel) and the true FDR (bottom left panel) and TPR (bottom right panel) for simulation study B_{625}^{625}. Figure 11 shows the corresponding figures for simulation study B_0^{4000}. We can see that already when the number of samples per condition is increased from 2 to 3, the FDR of most methods decrease noticeably, and the transformation-based methods (voom and vst) are able to detect differentially expressed genes. The non-parametric SAMseq method, however, requires more than 3 samples to be able to call differentially expressed genes at the imposed FDR cutoff.

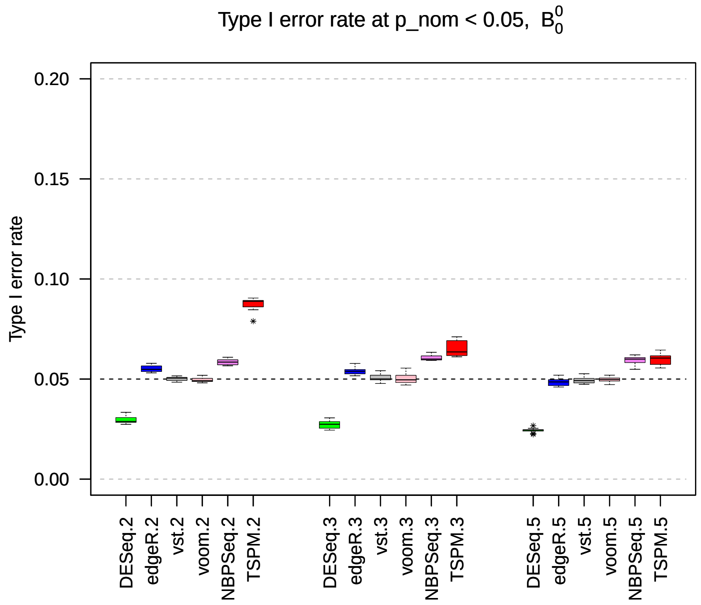

Supplementary Figure 9. *The type I error rate for simulation study* B_0^0 *at a nominal p-value threshold of 0.05, for 2, 3, and 5 samples per condition, respectively.*

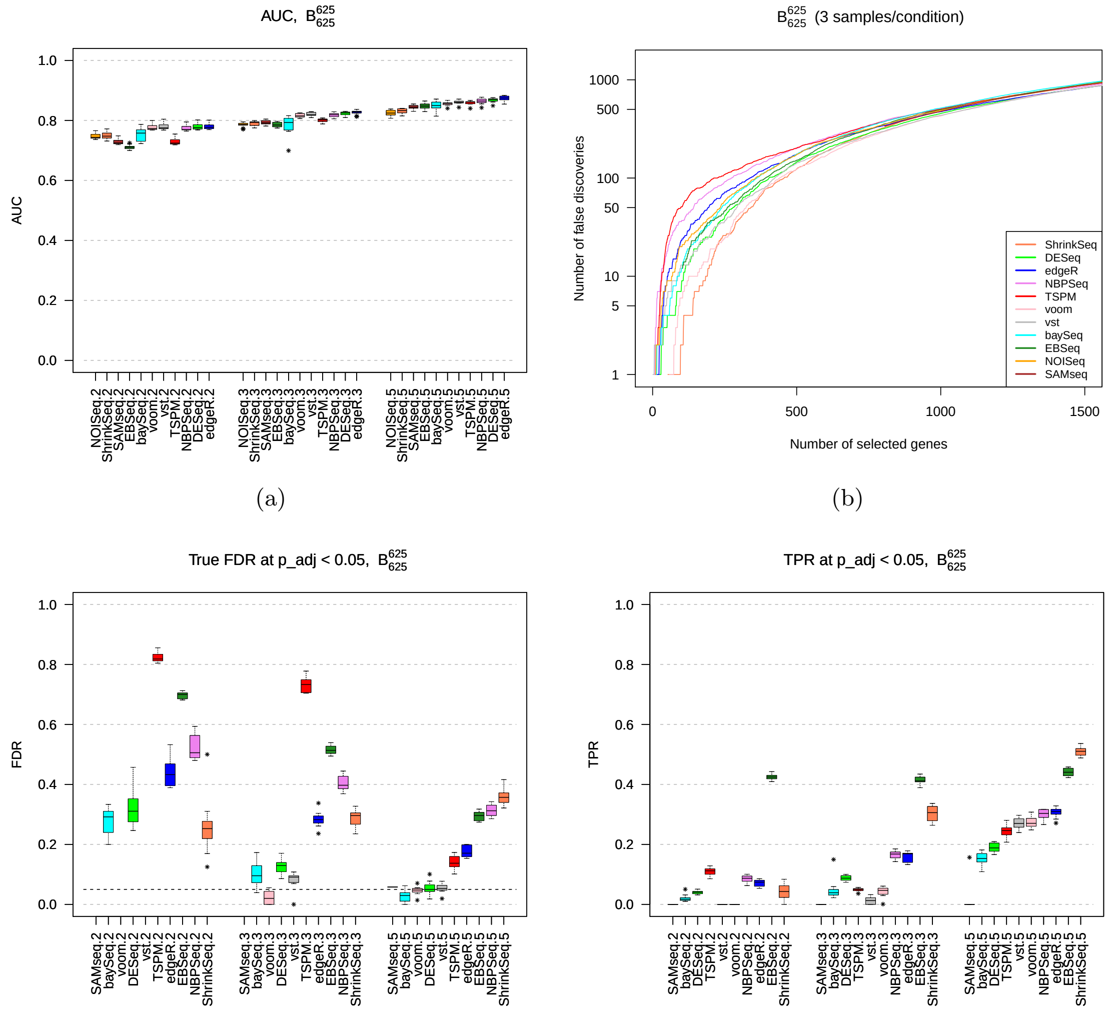

Supplementary Figure 10. *The AUC (a), representative false discovery curves (b), the observed FDR (c) and the observed TPR (d) for the 10 evaluated methods, in simulation study* B^{625}_{625}.

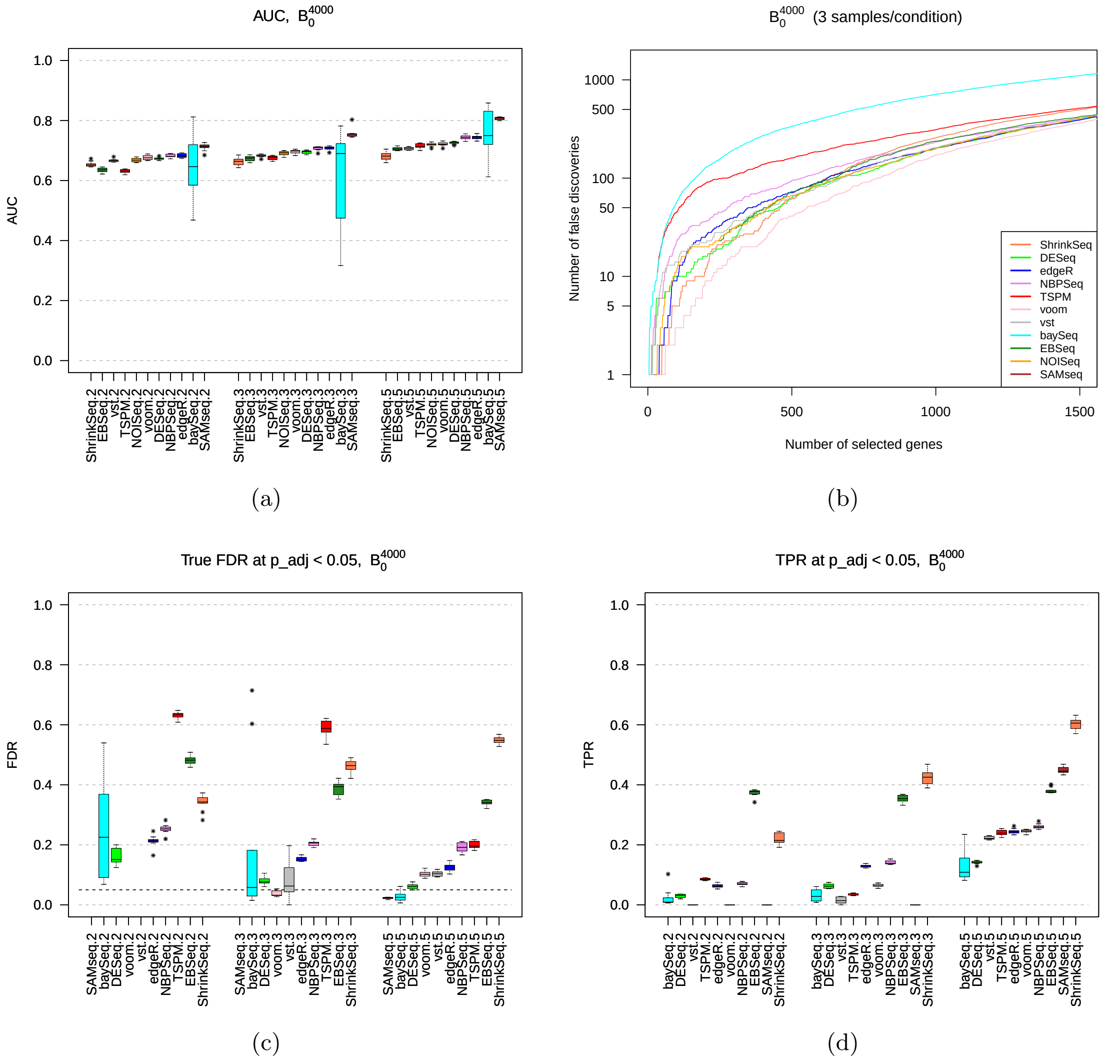

Supplementary Figure 11. *The AUC (a), representative false discovery curves (b), the observed FDR (c) and the observed TPR (d) for the 10 evaluated methods, in simulation study* B_0^{4000}.

6 Simulations with unequal dispersion across conditions

In the simulation results shown in the main article, we simulated the data using the same value for the dispersion (ϕ) in both compared conditions. In this section, we evaluate how the results are affected by having unequal dispersions in the two conditions. To generate data for these comparisons, we again make use of the mean-dispersion relationship estimated from real data (Supplementary Figure 1). As before, we draw the mean and dispersion parameter from condition S_1 from the estimated pairs. After computing the value of λ_{gS_2}, we then draw a corresponding value for ϕ_{gS_2} among the estimated dispersions in all pairs with a mean value similar to λ_{gS_2}. Hence, if gene g is not differentially expressed (so that $\lambda_{gS_1} = \lambda_{gS_2}$) we draw a dispersion value for gene g in condition S_2 among the dispersion estimates for genes with mean count close to λ_{gS_1}. If gene g is upregulated in S_2 compared to S_1, the dispersion will be drawn from a distribution which is somewhat shifted towards lower values, since the mean is higher. We will denote the simulation studies thus obtained by $B(\Delta\phi)$.

Figure 12 shows the type I error for simulation study $B(\Delta\phi)_0^0$, as well as the AUC and the observed FDR and TPR for simulation study $B(\Delta\phi)_{625}^{625}$. Figure 13 shows representative false discovery curves for simulation study $B(\Delta\phi)_{625}^{625}$, for 2, 5, and 10 samples per condition, respectively. The results in Supplementary Figure 12 can be compared to Figure 3A (for the type I error rate in simulation study B_0^0), Figure 1B (AUC in simulation study B_{625}^{625}), Figure 4B (FDR in simulation study B_{625}^{625}) and Supplementary Figure 22 (TPR for simulation study B_{625}^{625}). Similarly, the curves shown in Supplementary Figure 13 can be compared to Supplementary Figure 18, Figure 2B and Supplementary Figure 19. In general, the non-equal dispersions did not have a very large effect on the rankings of the genes. baySeq seemed to be somewhat negatively affected, and the transformation-based methods as well, while NOISeq appeared to be least affected. Larger effects were seen for the observed false discovery rates, where most methods performed worse when the dispersion differed between the conditions. The least affected methods were edgeR, NBPSeq and EBSeq. Notably, the FDR of voom+limma, vst+limma, SAMseq and baySeq, as well as the type I error rate of the transformation-based methods, increased considerably when the dispersions were different in the two conditions.

In fact, two of the methods considered here (DESeq and baySeq) allow the user to tune the parameters to obtain condition-specific dispersion estimates, which are then used in the differential expression analysis. Supplementary Figures 14 and 15 compare the results of using these settings to those obtained assuming that the dispersion is identical in the two groups. For DESeq, using the condition-specific dispersion estimates provides an advantage for the large sample sizes, but less so for very small sample sizes. Interestingly, using the condition-specific dispersion estimates seemed to worsen the performance of baySeq, especially for large sample sizes.

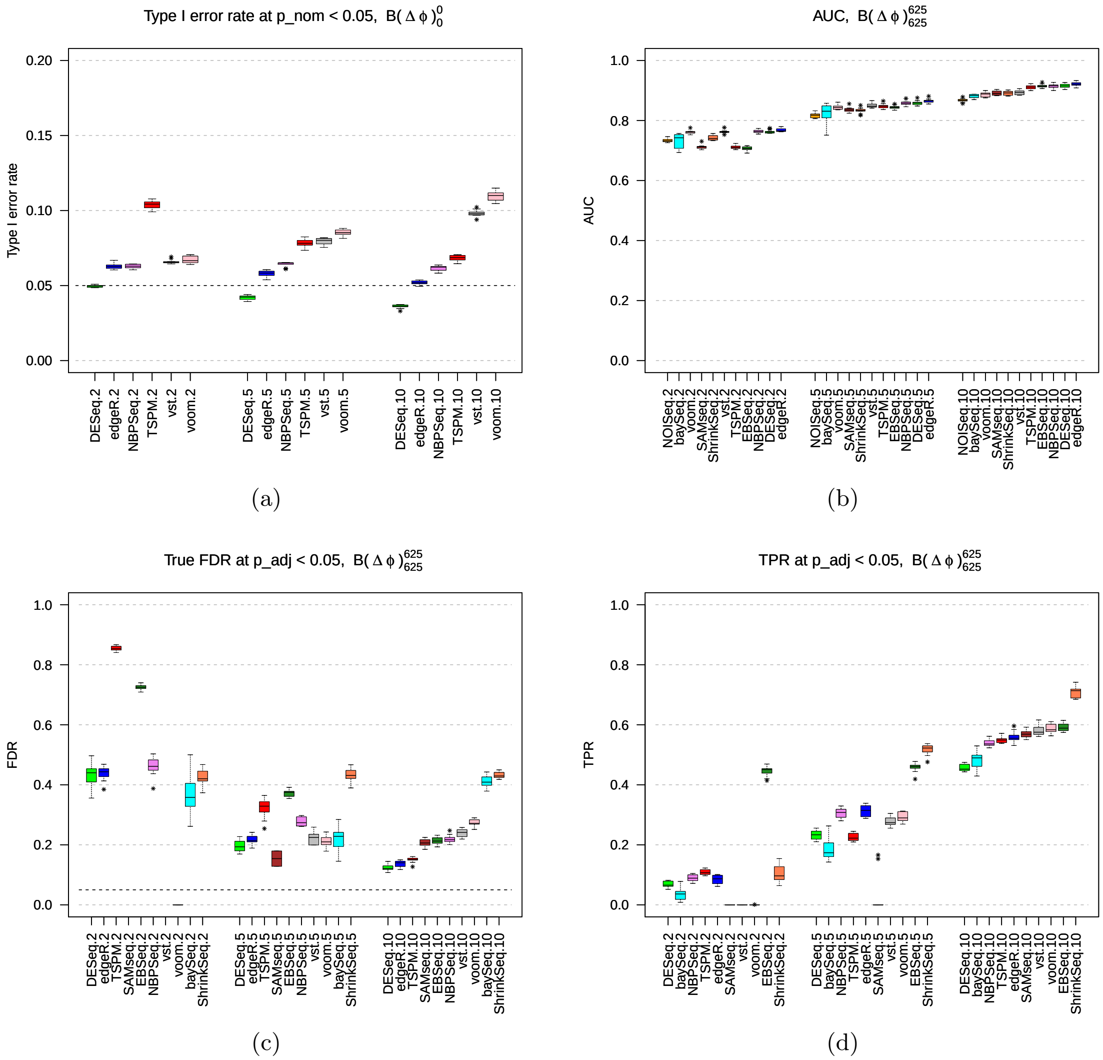

Supplementary Figure 12. *The type I error (a), AUC (b), the observed FDR (c) and the observed TPR (d) for the evaluated methods, in simulation studies* $B(\Delta\phi)_0^0$ *and* $B(\Delta\phi)_{625}^{625}$.

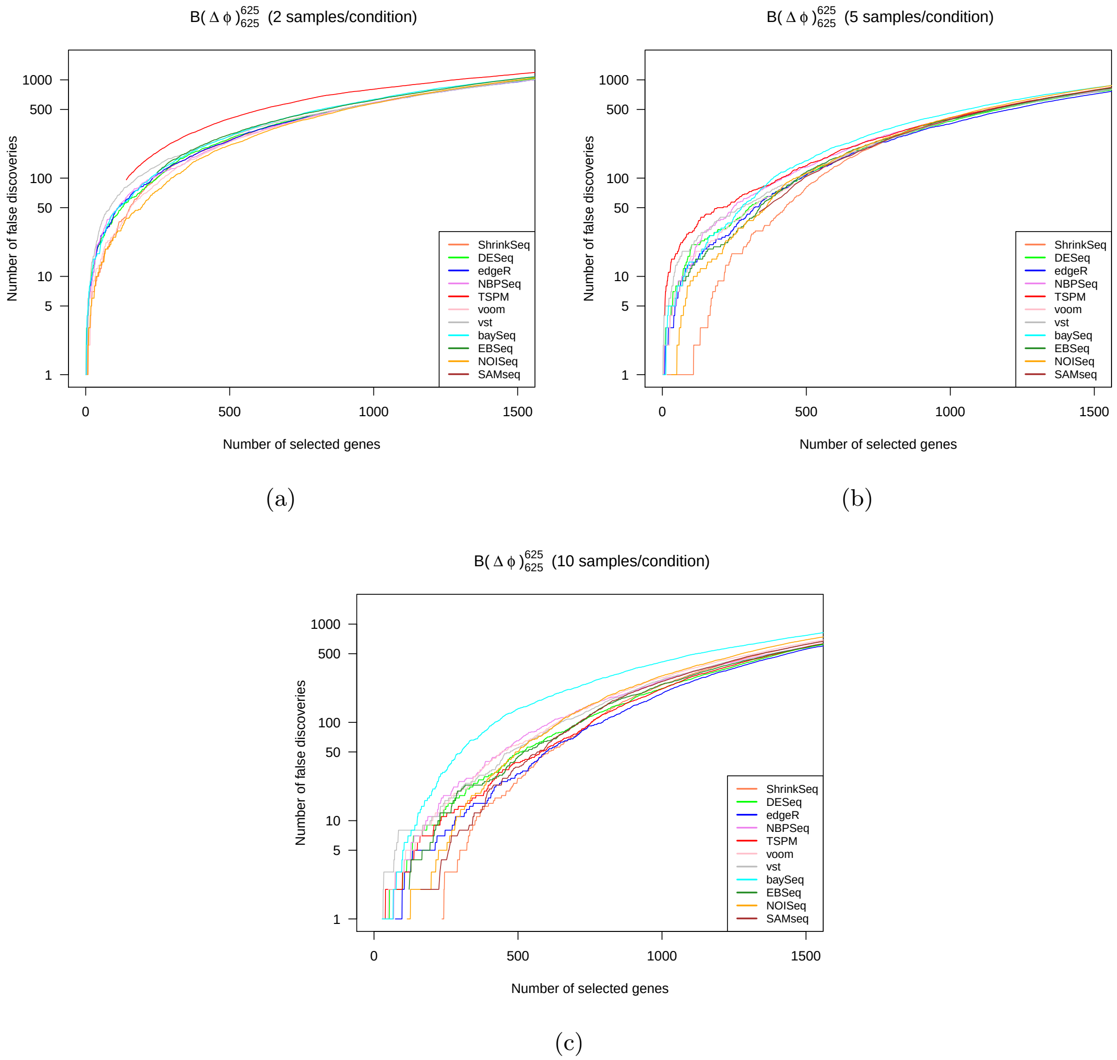

Supplementary Figure 13. *Representative false discovery curves for simulation study* $B(\Delta\phi)^{625}_{625}$*, for 2, 5, and 10 samples per condition, respectively.*

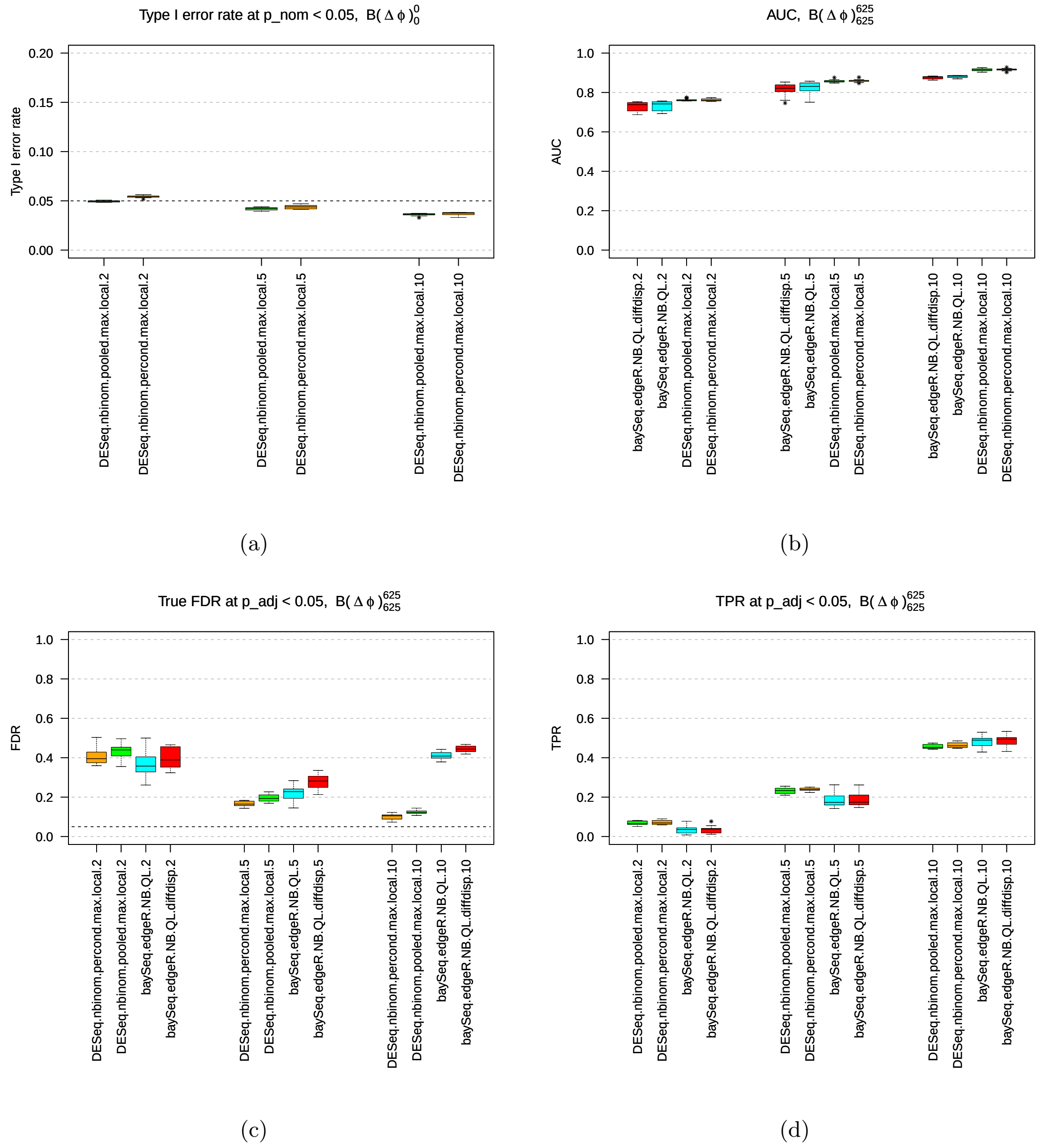

Supplementary Figure 14. *The type I error (a), AUC (b), the observed FDR (c) and the observed TPR (d) for DESeq and baySeq, with and without condition-specific dispersion estimates, in simulation studies* $B(\Delta\phi)_0^0$ *and* $B(\Delta\phi)_{625}^{625}$. *The methods denoted 'DESeq.nbinom.percond.max.local' and 'baySeq.edgeR.NB.QL.diffdisp' correspond to those estimating condition-specific dispersion parameters.*

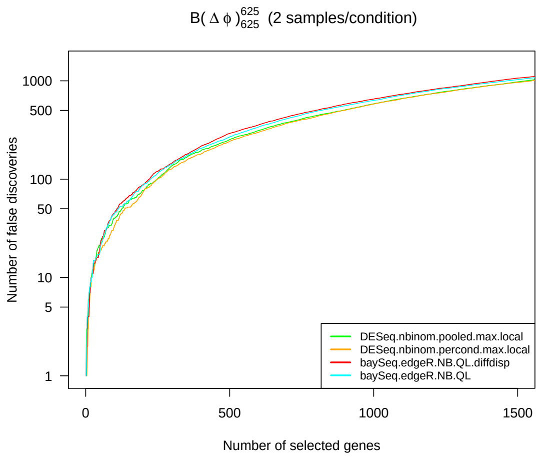

(a)

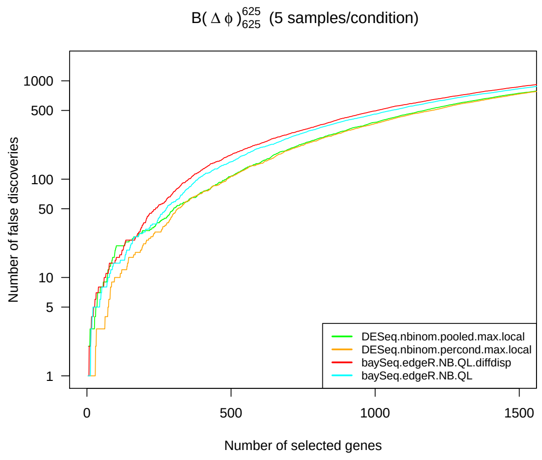

(b)

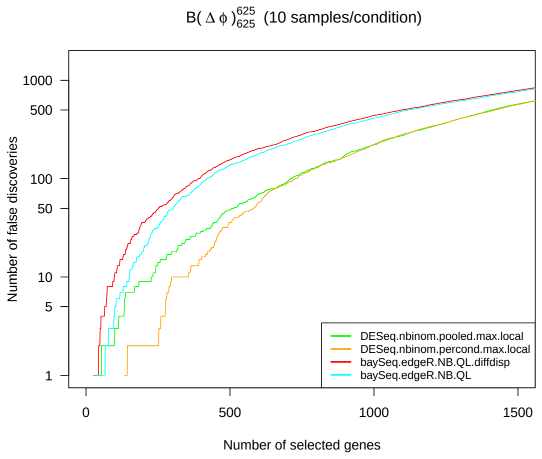

(c)

Supplementary Figure 15. *Representative false discovery curves for simulation study $B(\Delta\phi)^{625}_{625}$, for 2, 5, and 10 samples per condition, respectively, for DESeq and baySeq, with and without condition-specific dispersion estimates.*

7 R commands

Here, we give the R commands that were used to run the differential expression analyses in the main paper. For the analyses shown in the Supplementary Material, some parameter values were changed as indicated in the text (see also the help pages for the respective functions and packages). All analyses were performed with R version 2.15. We assume that we have a count matrix, denoted `count.matrix`, with $|G|$ rows (genes) and $|S|$ columns (samples). We also have a length-$|S|$ vector `class`, which encodes the conditions of the $|S|$ samples, represented as 1 and 2, respectively.

7.1 edgeR

The edgeR package can be installed from Bioconductor. In the present paper, we used version 2.7.11.

```
> library(edgeR)
> edgeR.dgelist = DGEList(counts = count.matrix, group = factor(class))
> edgeR.dgelist = calcNormFactors(edgeR.dgelist, method = "TMM")
> edgeR.dgelist = estimateCommonDisp(edgeR.dgelist)
> edgeR.dgelist = estimateTagwiseDisp(edgeR.dgelist, trend = "movingave")
> edgeR.test = exactTest(edgeR.dgelist)
> edgeR.pvalues = edgeR.test$table$PValue
> edgeR.adjpvalues = p.adjust(edgeR.pvalues, method = "BH")
```

7.2 DESeq

The DESeq package can be installed from Bioconductor. In the present paper, we used version 1.8.2.

```
> library(DESeq)
> DESeq.cds = newCountDataSet(countData = count.matrix,
+     conditions = factor(class))
> DESeq.cds = estimateSizeFactors(DESeq.cds)
> DESeq.cds = estimateDispersions(DESeq.cds, sharingMode = "maximum",
+     method = "pooled", fitType = "local")
> DESeq.test = nbinomTest(DESeq.cds, "1", "2")
> DESeq.pvalues = DESeq.test$pval
> DESeq.adjpvalues = p.adjust(DESeq.pvalues, method = "BH")
```

7.3 NBPSeq

The NBPSeq package can be installed from Bioconductor. In the present paper, we used version 0.1.6.

```
> library(edgeR)
> library(NBPSeq)
> NBPSeq.dgelist = DGEList(counts = count.matrix, group = factor(class))
```

```
> NBPSeq.dgelist = calcNormFactors(NBPSeq.dgelist, method = "TMM")
> NBPSeq.norm.factors = as.vector(NBPSeq.dgelist$samples$norm.factors)
> NBPSeq.test = nbp.test(counts = count.matrix, grp.ids = class,
+     grp1 = 1, grp2 = 2, norm.factors = NBPSeq.norm.factors,
+     method.disp = "NBP")
> NBPSeq.pvalues = NBPSeq.test$p.values
> NBPSeq.adjpvalues = NBPSeq.test$q.values
```

7.4 baySeq

The baySeq package can be installed from Bioconductor. In the present paper, we used version 1.10.0.

```
> library(baySeq)
> baySeq.cd = new("countData", data = count.matrix, replicates = class,
+     groups = list(NDE = rep(1, length(class)), DE = class))
> baySeq.cd@libsizes = getLibsizes(baySeq.cd, estimationType = "edgeR")
> baySeq.cd = getPriors.NB(baySeq.cd, samplesize = 5000,
+     equalDispersions = TRUE, estimation = "QL", cl = NULL)
> baySeq.cd = getLikelihoods.NB(baySeq.cd, prs = c(0.5,
+     0.5), pET = "BIC", cl = NULL)
> baySeq.posteriors.DE = exp(baySeq.cd@posteriors)[, 2]
> baySeq.table = topCounts(baySeq.cd, group = "DE", FDR = 1)
> baySeq.FDR = baySeq.table$FDR[match(rownames(count.matrix),
+     rownames(baySeq.table))]
```

7.5 EBSeq

The EBSeq package can be downloaded from `www.biostat.wisc.edu/~kendzior/EBSEQ/`. In the present paper, we used version 1.1.

```
> library(EBSeq)
> sizes = MedianNorm(count.matrix)
> EBSeq.test = EBTest(Data = count.matrix, Conditions = factor(class),
+     sizeFactors = sizes, maxround = 10)
> EBSeq.ppmat = GetPPMat(EBSeq.test)
> EBSeq.probabilities.DE = EBSeq.ppmat[, "PPDE"]
> EBSeq.lFDR = 1 - EBSeq.ppmat[, "PPDE"]
> EBSeq.FDR = rep(NA, length(EBSeq.lFDR))
> for (i in 1:length(EBSeq.lFDR)) {
+     EBSeq.FDR[i] = mean(EBSeq.lFDR[which(EBSeq.lFDR <=
+         EBSeq.lFDR[i])])
+ }
```

7.6 TSPM

The TSPM R script can be downloaded from `http://www.stat.purdue.edu/~doerge/software/TSPM.R`. The version used in this paper was downloaded on May 4, 2012.

```
> library(edgeR)
> source("TSPM.R")
> TSPM.dgelist = DGEList(counts = count.matrix, group = factor(class))
> TSPM.dgelist = calcNormFactors(TSPM.dgelist, method = "TMM")
> norm.lib.sizes = as.vector(TSPM.dgelist$samples$norm.factors) *
+     as.vector(TSPM.dgelist$samples$lib.size)
> TSPM.test = TSPM(counts = count.matrix, x1 = factor(class),
+     x0 = rep(1, length(class)), lib.size = norm.lib.sizes)
> TSPM.pvalues = TSPM.test$pvalues
> TSPM.adjpvalues = TSPM.test$padj
```

7.7 SAMseq

SAMseq is available from the `samr` package, which can be installed from CRAN. In the present paper, we used version 2.0.

```
> library(samr)
> SAMseq.test = SAMseq(count.matrix, class,
+     resp.type = "Two class unpaired",
+     geneid = rownames(count.matrix), genenames = rownames(count.matrix),
+     nperms = 100, nresamp = 20, fdr.output = 1)
> SAMseq.result.table = rbind(SAMseq.test$siggenes.table$genes.up,
+     SAMseq.test$siggenes.table$genes.lo)
> SAMseq.score = rep(0, nrow(count.matrix))
> SAMseq.score[match(SAMseq.result.table[,
+     1], rownames(count.matrix))] = as.numeric(SAMseq.result.table[,
+     3])
> SAMseq.FDR = rep(1, nrow(count.matrix))
> SAMseq.FDR[match(SAMseq.result.table[,
+     1], rownames(count.matrix))] = as.numeric(SAMseq.result.table[,
+     5])/100
```

7.8 NOISeq

The NOISeq R script can be downloaded from `http://bioinfo.cipf.es/noiseq/doku.php?id=downloads`. The version used in this paper was downloaded on May 2, 2012.

```
> library(edgeR)
> source("noiseq.r")
> nf = calcNormFactors(count.matrix)
> libsizes = apply(count.matrix, 2, sum)
> common.libsize = prod(libsizes^(1/length(libsizes)))
```

```
> normfactors = nf * libsizes/common.libsize
> norm.matrix = sweep(count.matrix, 2, normfactors, "/")
> NOISeq.test = noiseq(norm.matrix[, class == 1], norm.matrix[,
+     class == 2], repl = "bio", k = 0.5, norm = "n", long = 1000)
> NOISeq.probabilities = NOISeq.test$probab
```

7.9 voom+limma

The voom transformation is available within the limma package. which can be downloaded from Bioconductor. In the present paper, we used version 3.10.2.

```
> library(limma)
> nf = calcNormFactors(count.matrix, method = "TMM")
> voom.data = voom(count.matrix, design = model.matrix(~factor(class)),
+     lib.size = colSums(count.matrix) * nf)
> voom.data$genes = rownames(count.matrix)
> voom.fitlimma = lmFit(voom.data, design = model.matrix(~factor(class)))
> voom.fitbayes = eBayes(voom.fitlimma)
> voom.pvalues = voom.fitbayes$p.value[, 2]
> voom.adjpvalues = p.adjust(voom.pvalues, method = "BH")
```

7.10 vst+limma

The vst transformation is available within the DESeq package. In the present paper, we used version 1.8.2 of the DESeq package and version 3.10.2 of limma.

```
> library(DESeq)
> library(limma)
> DESeq.cds = newCountDataSet(countData = count.matrix,
+     conditions = factor(class))
> DESeq.cds = estimateSizeFactors(DESeq.cds)
> DESeq.cds = estimateDispersions(DESeq.cds, method = "blind",
+     fitType = "local")
> DESeq.vst = getVarianceStabilizedData(DESeq.cds)
> DESeq.vst.fitlimma = lmFit(DESeq.vst, design = model.matrix(~factor(class)))
> DESeq.vst.fitbayes = eBayes(DESeq.vst.fitlimma)
> DESeq.vst.pvalues = DESeq.vst.fitbayes$p.value[, 2]
> DESeq.vst.adjpvalues = p.adjust(DESeq.vst.pvalues, method = "BH")
```

7.11 ShrinkSeq

The ShrinkSeq is available as a function in the ShrinkBayes R package, which can be downloaded from `http://www.few.vu.nl/~mavdwiel/ShrinkBayes.html`. In this paper, we used version 1.6.

```
> library(ShrinkBayes)
> library(edgeR)
```

```
> nf = calcNormFactors(count.matrix, method = "TMM") *
+     colSums(count.matrix)/exp(mean(log(colSums(count.matrix))))
> count.matrix = round(sweep(count.matrix, 2, nf, "/"))
> group = factor(class)
> form = y ~ 1 + group
> ShrinkSeq.shrinkres = ShrinkSeq(form = form, dat = count.matrix,
+     shrinkfixed = "group", mixtdisp = FALSE, shrinkdisp = TRUE,
+     fams = "zinb", ncpus = 1)
> ShrinkSeq.fitzinb = FitAllShrink(forms = form, dat = count.matrix,
+     fams = "zinb", shrinksimul = ShrinkSeq.shrinkres,
+     ncpus = 1)
> ShrinkSeq.npprior = NonParaUpdatePrior(fitall = ShrinkSeq.fitzinb,
+     modus = "fixed", shrinkpara = "group", maxiter = 15,
+     ncpus = 1)
> ShrinkSeq.nppostshr = NonParaUpdatePosterior(ShrinkSeq.fitzinb,
+     ShrinkSeq.npprior, ncpus = 1)
> ShrinkSeq.lfdrless = SummaryWrap(ShrinkSeq.nppostshr,
+     thr = 0, direction = "lesser")
> ShrinkSeq.lfdrgreat = SummaryWrap(ShrinkSeq.nppostshr,
+     thr = 0, direction = "greater")
> ShrinkSeq.FDR = BFDR(ShrinkSeq.lfdrless, ShrinkSeq.lfdrgreat)
```

8 Computational time requirement

To estimate the computational time required to find DE genes with each of the evaluated methods, we ran the code shown in the previous section to perform the differential expression analysis for each method, and measured the required CPU time. The analysis were performed on one instance of simulation study B_{625}^{625} for each sample size (2, 5, and 10 samples per condition, respectively). The number of genes remaining after filtering out those with fewer than 10 counts in total were, respectively, 12,068 (2 samples per condition), 12,412 (5 samples per condition) and 12,490 (10 samples per condition). The computational time for some methods depends heavily on parameter choices, such as the number of iterations or resamplings and whether or not a cluster is used, and the resulting estimates, presented in Supplementary Figure 16, therefore merely reflect the relative time requirements between the methods with the parameter values set as in the present study.

The transformation-based methods (voom+limma and vst+limma) required the least computational time, while baySeq and ShrinkSeq were by far the slowest with the current settings. However, both these methods can be parallelized. The time requirements for the non-parametric methods (SAMseq and NOISeq), as well as for EBSeq, were highly dependent on the sample size. On the contrary, the computational times required for edgeR, TSPM, ShrinkSeq and the transformation-based methods were largely unaffected by varying the sample size within the range used in the present study.

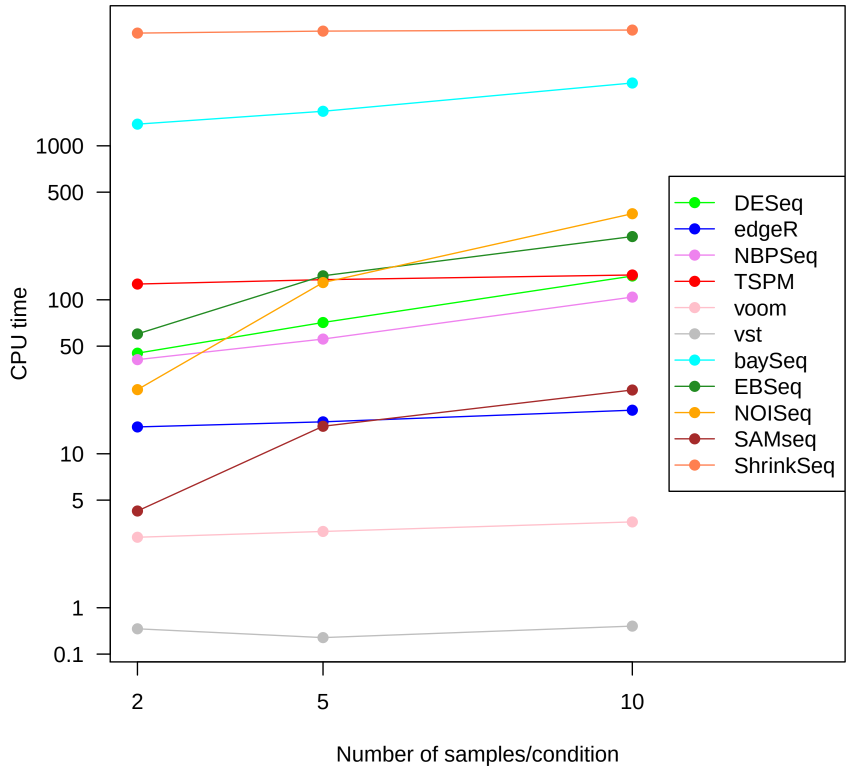

Supplementary Figure 16. *CPU time required to run the differential expression analysis for each of the evaluated methods, for different sample sizes. The evaluation was based on one instance of simulation study* B_{625}^{625} *for each sample size. The code that was timed is given in Section 7.*

9 Supplementary Figures

The following section contains supplementary figures referred to in the main article. We use the same parameter values as in the main article. Supplementary Figure 17 shows the results obtained for simulation study P_{625}^{625}. Letting the counts for some genes follow a Poisson distribution rather than a Negative Binomial distribution increased the AUC and TPR for all methods and decreased the FDR, most notably for TSPM and EBSeq.

Supplementary Figures 18 and 19 shows representative false discovery curves for all methods, with 2 and 10 samples per condition, respectively, in different simulation studies. These curves can be compared to Figure 2 in the main paper, where we show the results for 5 samples per condition. It is clear from these figures that increasing the number of samples leads to fewer false discoveries among the top-ranked genes.

Supplementary Figures 20 and 21 show representative p-value distributions for the six methods returning nominal p-values, in all four simulation studies without any truly DE genes. When we introduced outliers with abnormally high counts (simulation studies S_0^0 and R_0^0, Supplementary Figure 21), edgeR and NBPSeq were clearly becoming more liberal (more low p-values) while DESeq was oppositely affected. TSPM experienced an enrichment of p-values between 0.05 and 0.3, while the two transformation-based methods (voom+limma and vst+limma) were less affected. Letting the counts for half of the genes follow a Poisson distribution (simulation study P_0^0, Supplementary Figure 20(b)) depleted the medium-sized p-values for the three methods relying on a NB distribution (edgeR, DESeq and NBPSeq), but did not affect the fraction of p-values below 0.05 much. The p-value distributions for the other three methods were only marginally affected.

In Supplementary Figure 22, we show the true positive rates (TPR) among the genes with adjusted p-value below 0.05, for the different simulation studies. These figures should be interpreted in conjunction with Figure 4 in the main article, which shows the corresponding FDRs.

Supplementary Figure 23 shows MA-type plots for all genes in the Bottomly data set. To generate the plots, the counts were first normalized using normalization factors computed by the TMM method combined with the observed library sizes. We then added 0.5 to all normalized counts and log-transformed the result. On the x-axis, we plotted the average of the mean values in the two conditions, and on the y-axis we plotted the difference between the same values. Hence, the x-axis depicts a measure of the overall expression level of the genes, and the y-axis shows a measure of the level of differential expression between the two conditions. The colored dots are the genes that are called DE by the respective methods.

In Supplementary Figures 24-28, we show the genes identified as DE by only one method in the Bottomly data set. For methods identifying more than nine unique DE genes, only nine are shown. Finally, in Supplementary Figure 29 we show the ranking scores obtained for the Bottomly data set, by the eleven evaluated methods. We also include the rank correlations between the ranking scores for each pair of methods.

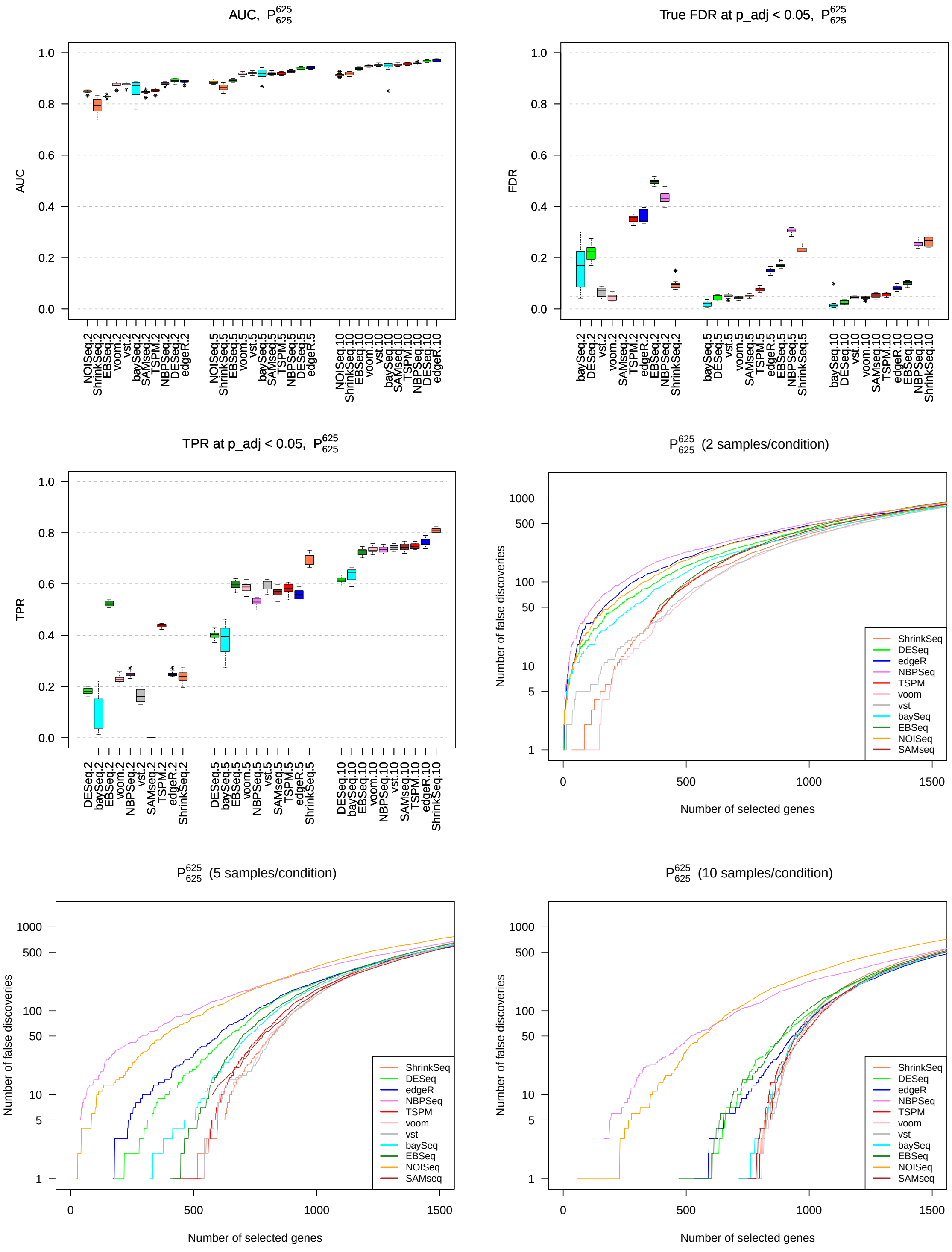

Supplementary Figure 17. *Results for the P^{625}_{625} simulation study.*

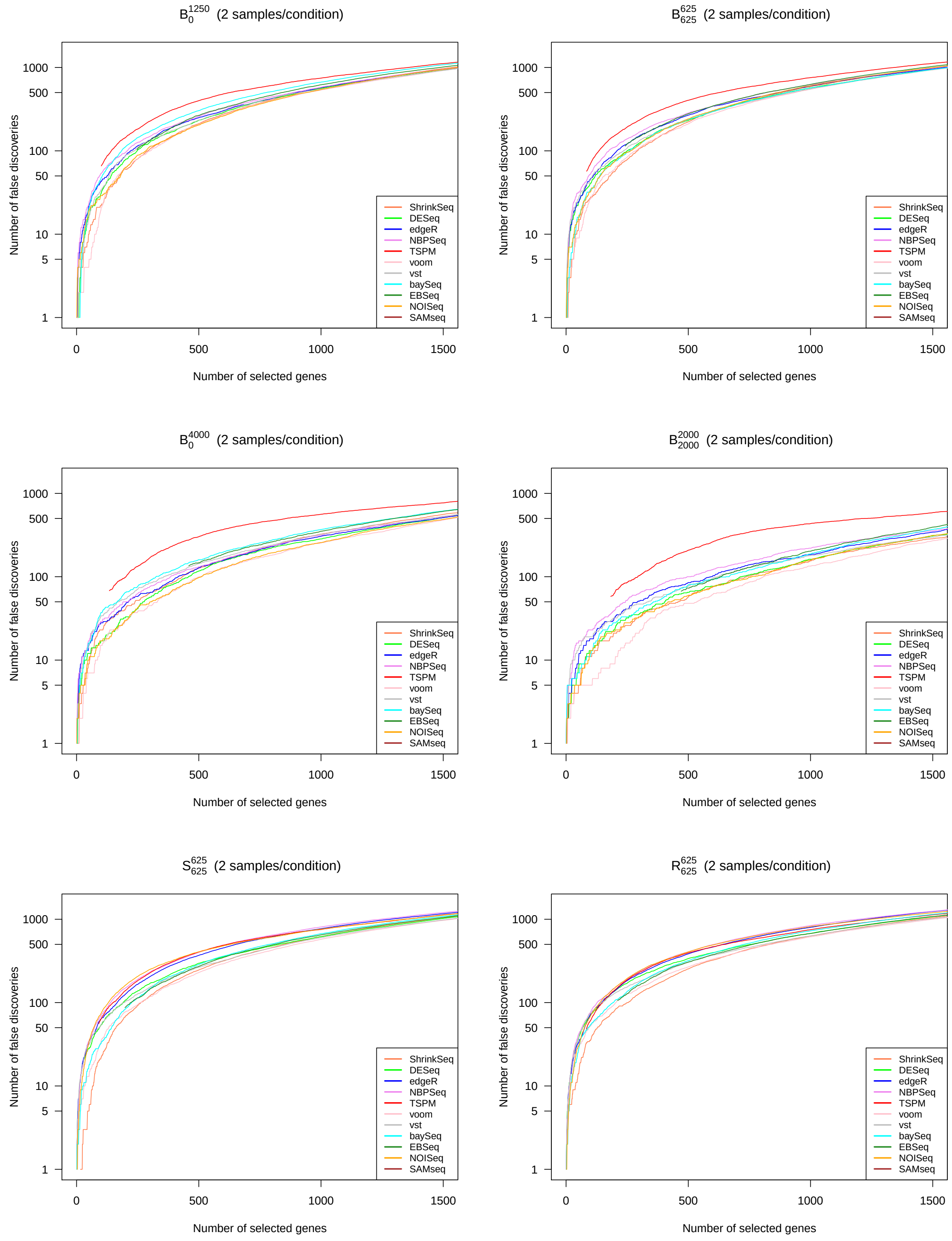

Supplementary Figure 18. *False discovery curves for the evaluated methods in different simulation studies, with 2 samples per condition.*

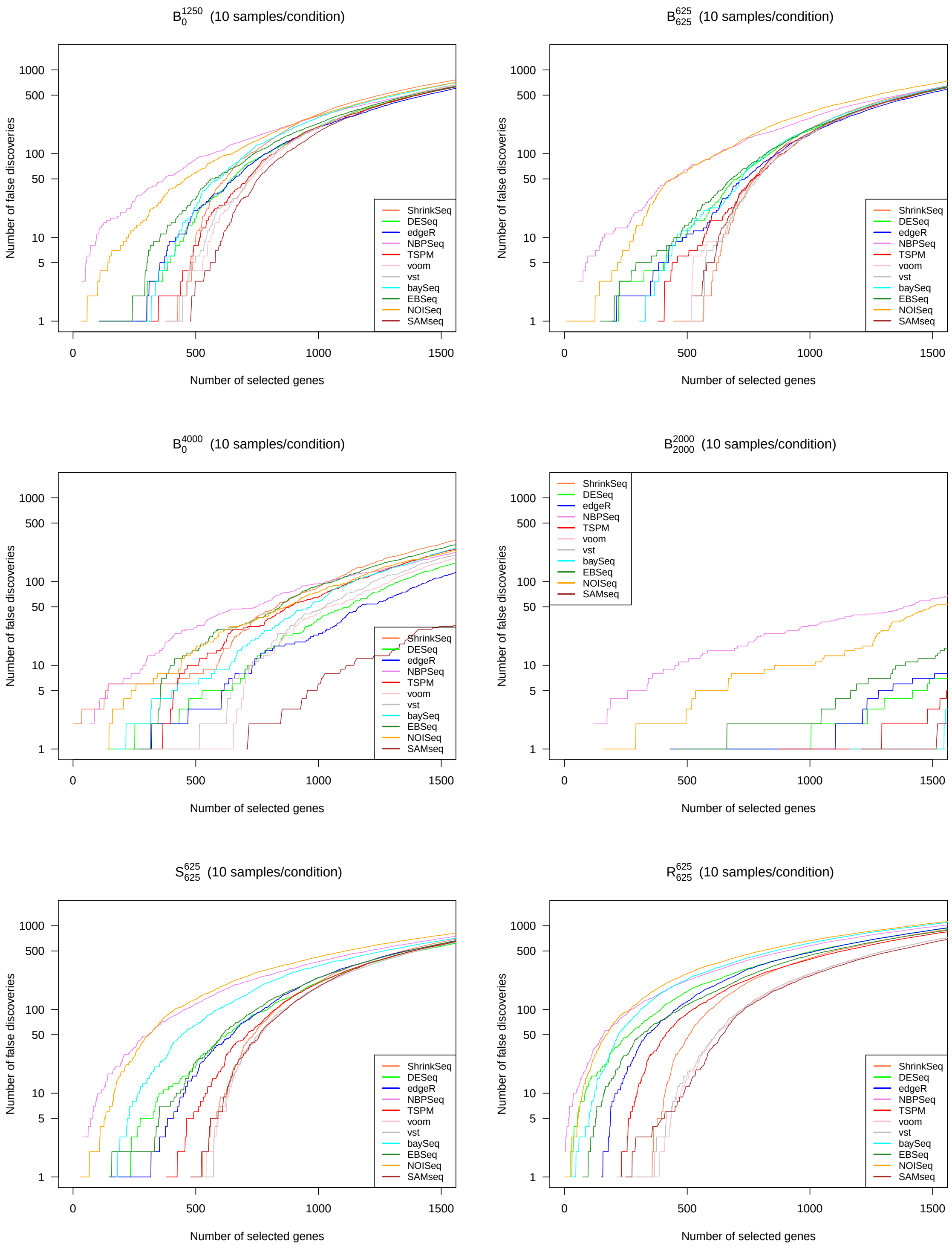

Supplementary Figure 19. *False discovery curves for the evaluated methods in different simulation studies, with 10 samples per condition.*

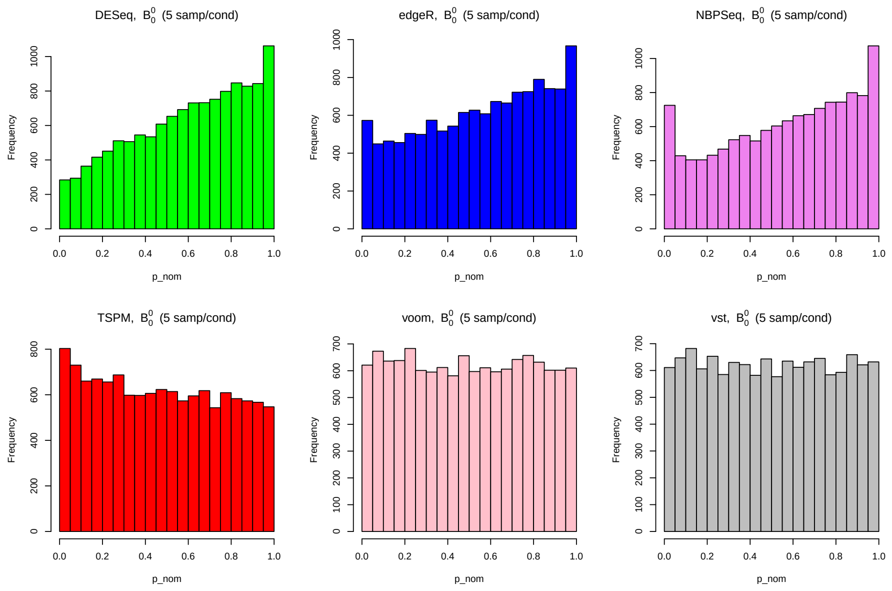

(a)

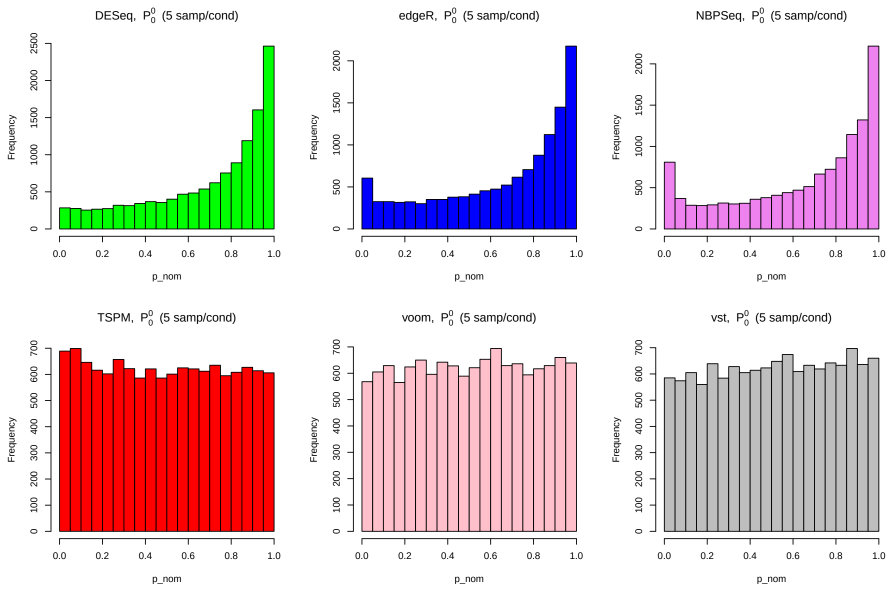

(b)

Supplementary Figure 20. *Representative p-value distributions obtained by the six methods returning nominal p-values, for data sets generated according to simulation studies B_0^0 (panel (a)) and P_0^0 (panel (b)) with 5 samples per condition.*

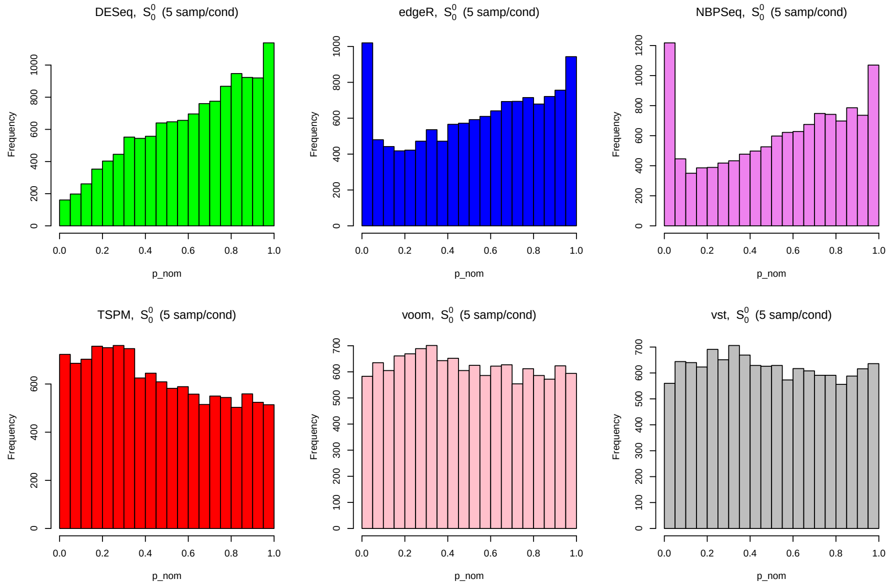

(a)

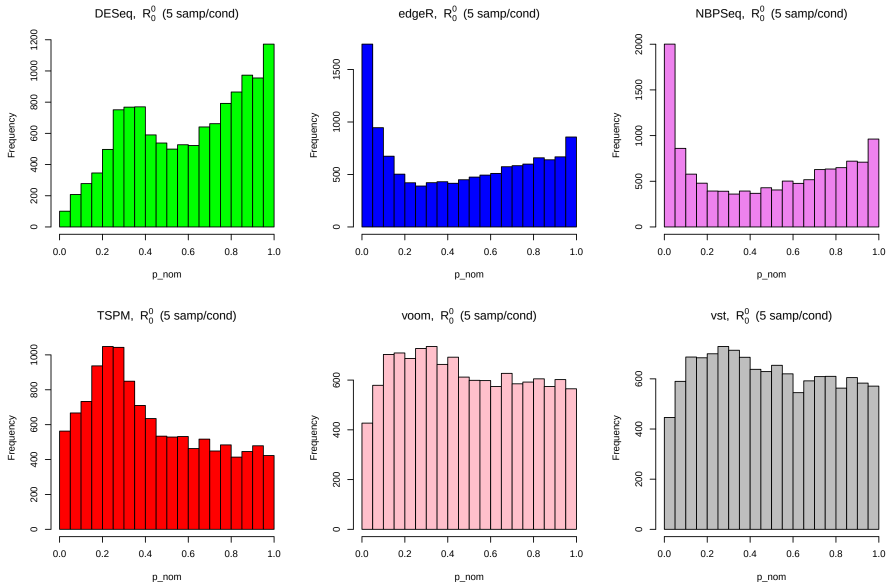

(b)

Supplementary Figure 21. *Representative p-value distributions obtained by the six methods returning nominal p-values, for data sets generated according to simulation studies S_0^0 (panel (a)) and R_0^0 (panel (b)) for 5 samples per condition.*

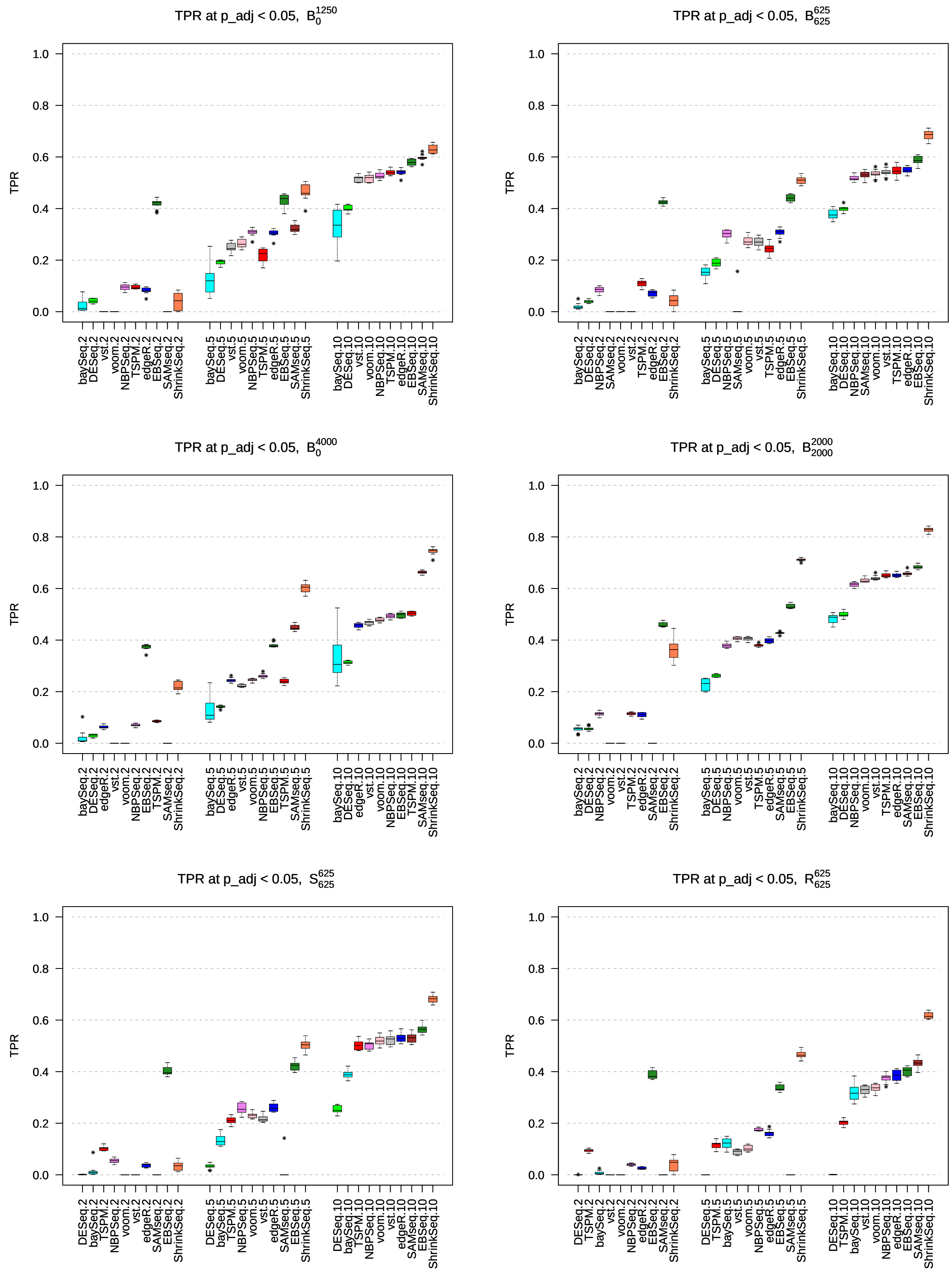

Supplementary Figure 22. *Observed TPRs for the evaluated methods across different simulation studies.*

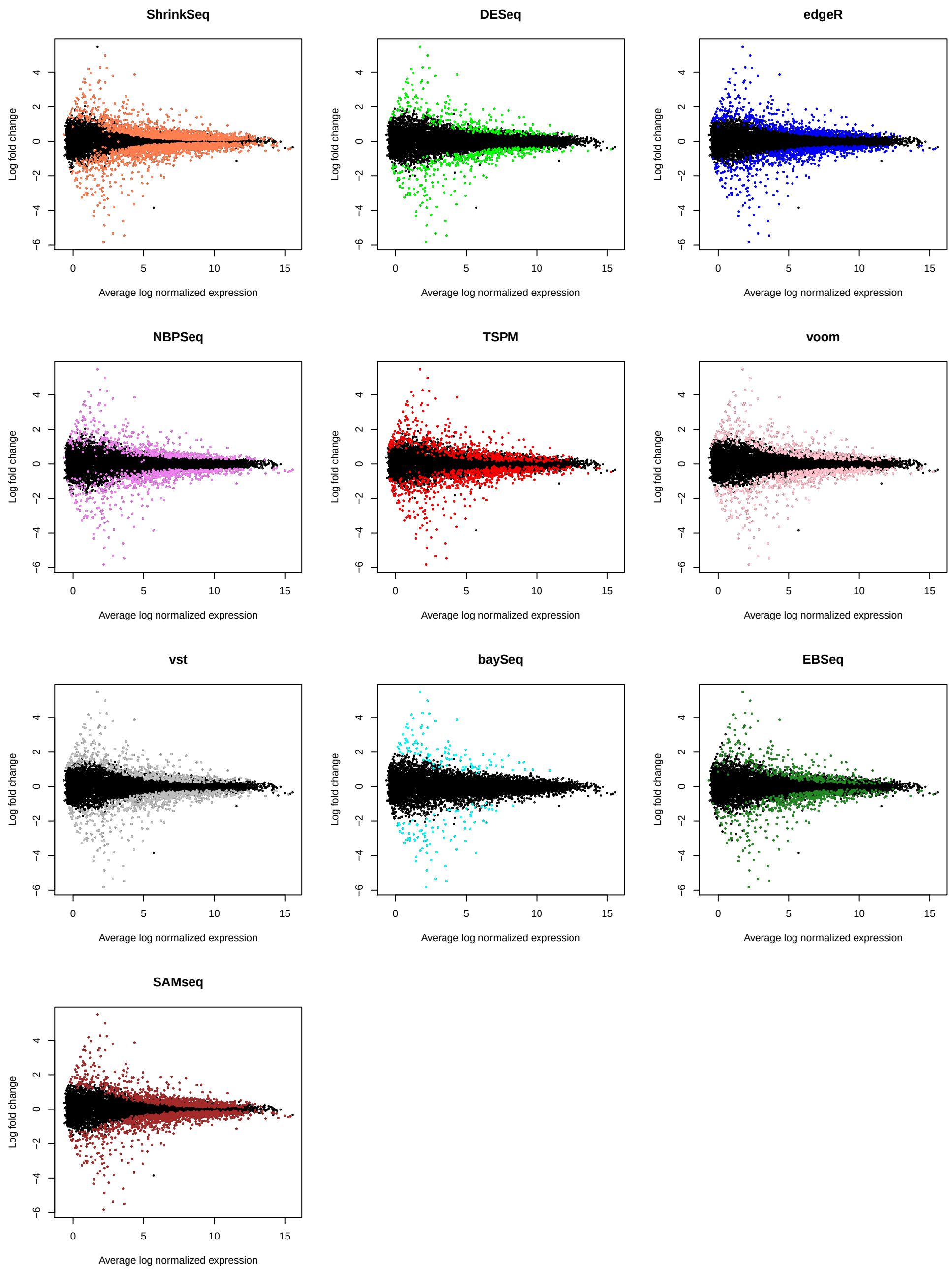

Supplementary Figure 23. *MA-type plots for the Bottomly data set, depicting the expression level (on the x-axis) and the level of differential expression between the two conditions (on the y-axis), with the genes called DE by the different methods indicated with color.*

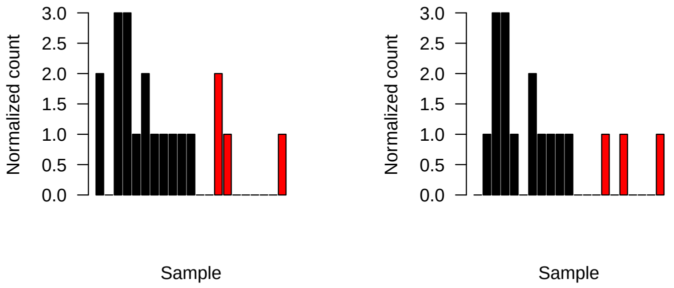

Supplementary Figure 24. *The two DE genes found exclusively by vst+limma, for the Bottomly data set.*

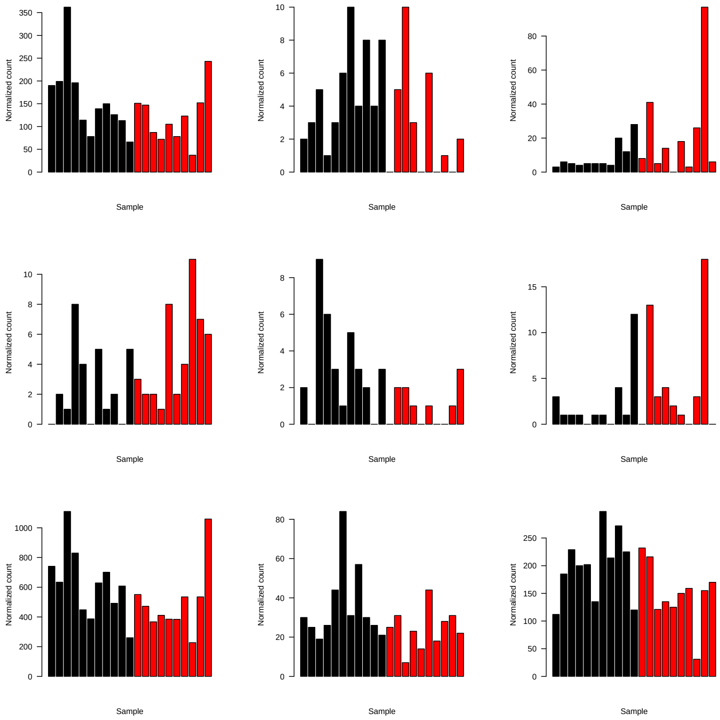

Supplementary Figure 25. *Nine of the DE genes found exclusively by NBPSeq, for the Bottomly data set.*

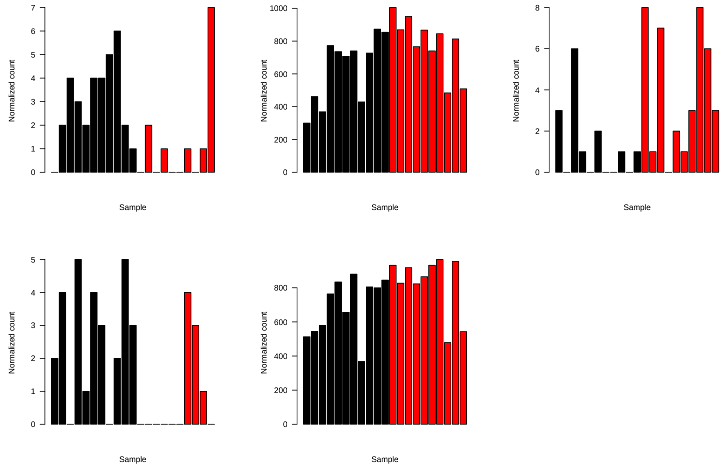

Supplementary Figure 26. *The five DE genes found exclusively by SAMseq, for the Bottomly data set.*

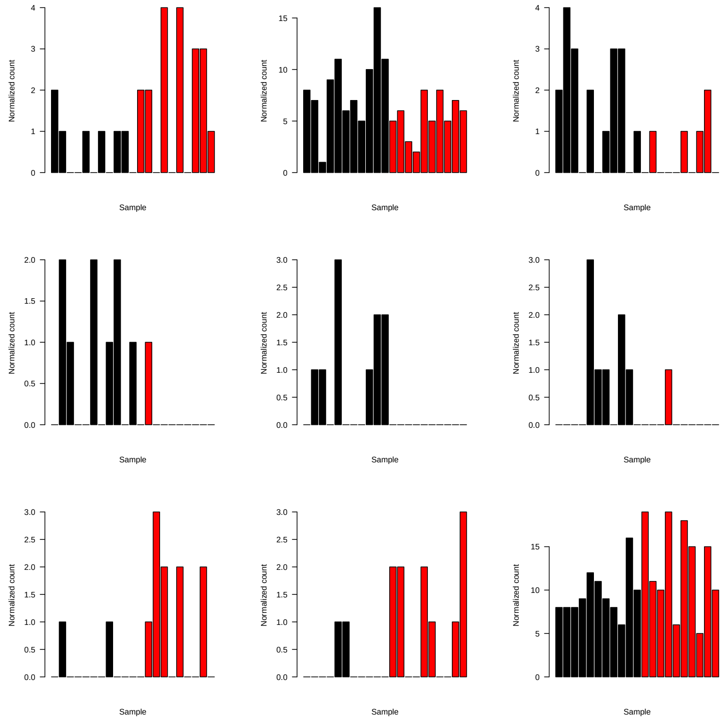

Supplementary Figure 27. *Nine of the DE genes found exclusively by TSPM, for the Bottomly data set.*

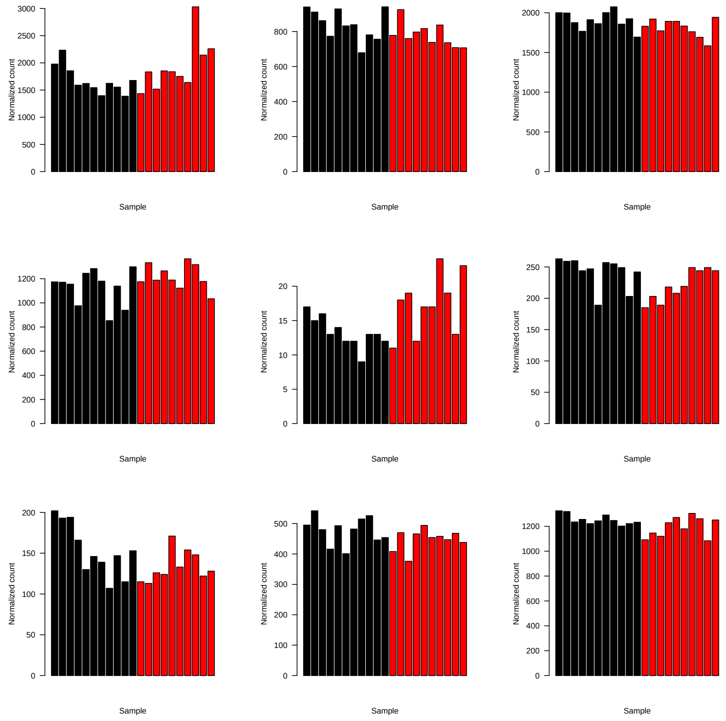

Supplementary Figure 28. *Nine of the DE genes found exclusively by ShrinkSeq, for the Bottomly data set.*

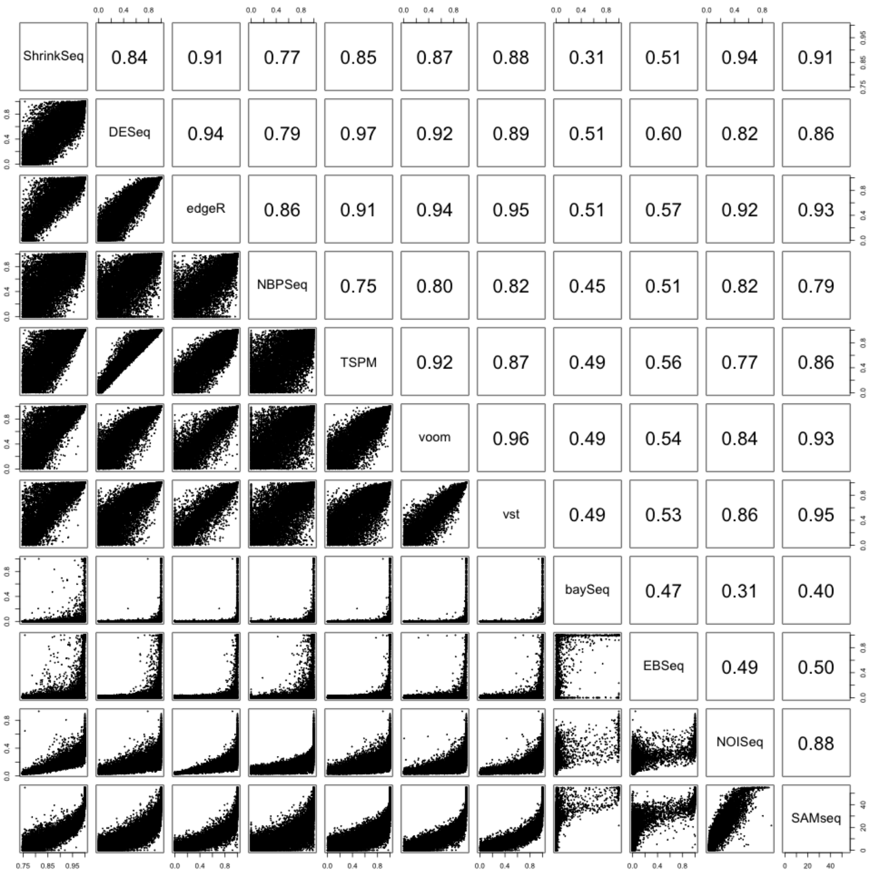

Supplementary Figure 29. *Relationships between the gene ranking scores obtained by the different methods for the Bottomly data set. The numbers above the diagonal are the Spearman rank correlations.*

References

[1] S Anders and W Huber. Differential expression analysis for sequence count data. *Genome Biology*, 11:R106, 2010.

[2] R Blekhman, J C Marioni, P Zumbo, M Stephens, and Y Gilad. Sex-specific and lineage-specific alternative splicing in primates. *Genome Research*, 20(2):180–189, 2010.

[3] J H Bullard, E Purdom, K D Hansen, and S Dudoit. Evaluation of statistical methods for normalization and differential expression in mRNA-Seq experiments. *BMC Bioinformatics*, 11:94, 2010.

[4] V G Cheung, R R Nayak, I X Wang, S Elwyn, S M Cousins, M Morley, and R S Spielman. Polymorphic cis- and trans-regulation of human gene expression. *PLoS Biology*, 8(9):e1000480, 2010.

[5] A C Frazee, B Langmead, and J T Leek. ReCount: a multi-experiment resource of analysis-ready RNA-seq gene count datasets. *BMC Bioinformatics*, 12:449, 2011.

[6] P Hammer, M S Banck, R Amberg, C Wang, G Petznick, S Luo, I Khrebtukova, G P Schroth, P Beyerlein, and A S Beutler. mRNA-seq with agnostic splice site discovery for nervous system transcriptomics tested in chronic pain. *Genome Research*, 20(6):847–860, 2010.

[7] D J McCarthy, Y Chen, and G K Smyth. Differential expression analysis of multifactor RNA-Seq experiments with respect to biological variation. *Nucleic Acids Research*, 40(10):4288–4297, 2012.

[8] J K Pickrell, J C Marioni, A A Pai, J F Degner, B E Engelhardt, E Nkadori, J B Veyrieras, M Stephens, Y Gilad, and J K Pritchard. Understanding mechanisms underlying human gene expression variation with RNA sequencing. *Nature*, 464:768–772, 2010.

[9] M D Robinson, D J McCarthy, and G K Smyth. edgeR: a Bioconductor package for differential expression analysis of digital gene expression data. *Bioinformatics*, 26:139–140, 2010.

[10] M D Robinson and A Oshlack. A scaling normalization method for differential expression analysis of RNA-seq data. *Genome Biology*, 11:R25, 2010.

[11] M D Robinson and G K Smyth. Small-sample estimation of negative binomial dispersion, with applications to SAGE data. *Biostatistics*, 9:321–332, 2008.

[12] J A Robles, S E Qureshi, S J Stephen, S R Wilson, C J Burden, and J M Taylor. Efficient experimental design and analysis strategies for the detection of differential expression using RNA-sequencing. 2012. http://dayhoff.anu.edu.au/RNA-SeqMultiplexPaper.pdf.
